# Ai-lncRNA *EGOT* enhancing autophagy sensitizes paclitaxel cytotoxicity via upregulation of ITPR1 expression by RNA-RNA and RNA-protein interactions in human cancer

**DOI:** 10.1186/s12943-019-1017-z

**Published:** 2019-04-18

**Authors:** Shouping Xu, Peiyuan Wang, Jian Zhang, Hao Wu, Shiyao Sui, Jinfeng Zhang, Qin Wang, Kun Qiao, Weiwei Yang, Hongbiao Xu, Da Pang

**Affiliations:** 1 Department of Breast Surgery, Harbin Medical University Cancer Hospital, 150 Haping Road, Harbin, 150081 China; 2 Department of Pathology, Harbin Medical University, Harbin, China; 3 Heilongjiang Academy of Medical Sciences, 157 Baojian Road, Harbin, 150086 China

**Keywords:** Ai-lncRNA, *EGOT*, ITPR1, Paclitaxel, Autophagy, Cancer

## Abstract

**Background:**

The biology function of antisense intronic long noncoding RNA (Ai-lncRNA) is still unknown. Meanwhile, cancer patients with paclitaxel resistance have limited therapeutic options in the clinic. However, the potential involvement of Ai-lncRNA in paclitaxel sensitivity remains unclear in human cancer.

**Methods:**

Whole transcriptome sequencing of 33 breast specimens was performed to identify Ai-lncRNA *EGOT*. Next, the role of *EGOT* in regulation of paclitaxel sensitivity was investigated. Moreover, the mechanism of *EGOT* enhancing autophagy sensitizes paclitaxel cytotoxicity via upregulation of ITPR1 expression by RNA-RNA and RNA-protein interactions was investigated in detail. Furthermore, upstream transcriptional regulation of *EGOT* expression was also investigated by co-immunoprecipitation and chromatin immunoprecipitation. Finally, clinical breast specimens in our cohort, TCGA and ICGC were applied to validate the role of *EGOT* in enhancing of paclitaxel sensitivity.

**Results:**

*EGOT* enhances autophagosome accumulation via the up-regulation of ITPR1 expression, thereby sensitizing cells to paclitaxel toxicity. Mechanistically, on one hand, *EGOT* upregulates ITPR1 levels via formation of a *pre-ITPR1/EGOT* dsRNA that induces *pre-ITPR1* accumulation to increase ITPR1 protein expression *in cis*. On the other hand, *EGOT* recruits hnRNPH1 to enhance the alternative splicing of pre-ITPR1 *in trans* via two binding motifs in *EGOT* segment 2 (324–645 nucleotides) in exon 1. Moreover, *EGOT* is transcriptionally regulated by stress conditions. Finally, *EGOT* expression enhances paclitaxel sensitivity via assessment of cancer specimens.

**Conclusions:**

These findings broaden comprehensive understanding of the biology function of Ai-lncRNAs. Proper regulation of *EGOT* may be a novel synergistic strategy for enhancing paclitaxel sensitivity in cancer therapy.

**Electronic supplementary material:**

The online version of this article (10.1186/s12943-019-1017-z) contains supplementary material, which is available to authorized users.

## Background

The microtubule-disrupting agent paclitaxel, a plant alkaloid developed from the bark of the Pacific yew tree, *Taxus brevifolia*, is a first-line chemotherapeutic agent for solid tumors, such as lung, ovarian and breast cancer, and contributes to substantial improvement in patient survival [1]. However, clinical responses to paclitaxel have shown variable sensitivity in cancer patients [2]. Thus, elucidation of the underlying mechanisms of paclitaxel sensitivity and identification of reliable biomarkers that can predict the response to paclitaxel in human cancer are urgently required.

Autophagy is a homeostatic process for degrading cellular materials under cellular stress [3]. Accumulating evidence has shown that autophagy is involved in modulating paclitaxel sensitivity. Tumors expressing diminished levels of autophagy-initiating genes are resistant to paclitaxel therapy, whereas induction of autophagy improves the antitumoral efficiency of the microtubule-disrupting agent paclitaxel [4, 5]. Furthermore, several studies have proposed that autophagy-related genes/proteins could be investigated as possible prognostic or predictive markers of paclitaxel efficacy in the clinic [6]. However, it is not clear which autophagy-initiating genes can be used as a predictive factor for paclitaxel response.

Long noncoding RNAs (lncRNAs) are a heterogeneous class of transcripts with a minimum length of 200 bases and limited protein-coding potential [7]. LncRNAs exhibit a wide range of expression levels and distinct cellular localizations and are thus a large and diverse class of regulators [8]. LncRNAs can function *in cis* to regulate the expression of neighboring genes or *in trans* to perform many roles in various modes [9]. The landscape of lncRNAs dysregulated in autophagy and their molecular mechanisms in autophagy regulatory networks were summarized in our previous study in detail [10]. Studies have demonstrated that lncRNAs such as *NBR2* and *APF* can regulate autophagy processes with the aid of certain important proteins [10]. Several lncRNAs have also recently been implicated in the modulation of drug resistance [11]. However, the relationship between lncRNA expression and paclitaxel insensitivity caused by abnormal autophagy remains largely unexplored.

Eosinophil granule ontogeny transcript (*EGOT*), as an antisense intronic long noncoding RNA (Ai-lncRNA), is expressed from *ITPR1*, which is a ligand-gated ion channel that mediates calcium release from intracellular stores [12, 13]. The biology function of Ai-lncRNAs including *EGOT* is still largely unknown. In our previous study, we first reported that downregulation of *EGOT* expression was correlated with advanced malignant status and worse prognosis in breast cancer [14]. Here, we describe a previously unrecognized role of *EGOT* in increasing sensitivity to paclitaxel via triggering autophagy. Mechanistically, *EGOT* enhances autophagosome accumulation via the upregulation of ITPR1 expression *in cis* and *in trans*. On one hand, *EGOT* upregulates ITPR1 levels via formation of a *pre-ITPR1/EGOT* dsRNA that induces *pre-ITPR1* accumulation to increase ITPR1 protein expression *in cis*. On the other hand, *EGOT* recruits hnRNPH1 to enhance the alternative splicing of pre-ITPR1 *in trans* via two binding motifs in *EGOT* segment 2 (324–645 nucleotides) in exon 1. We also uncover that hypoxia induces *EGOT* transcriptional expression, while estrogen suppresses its expression directly. Finally, *EGOT* is confirmed to be associated with a favorable prognosis and to enhance paclitaxel sensitivity in breast cancer patients and other human malignancies in our breast cancer cohort and public data cohorts. Given its significance in the autophagy signaling pathway, *EGOT* may be act as a promising predictive biomarker for paclitaxel response, and proper regulation of *EGOT* may be a novel synergistic strategy for enhancing paclitaxel sensitivity in human cancer.

## Materials and methods

### Breast specimens and clinical assessments

Eligible patients with a histological diagnosis of breast cancer who had received neither chemotherapy nor radiotherapy before surgical resection were recruited to the present study. In total, 258 breast cancer tissues and 258 normal tissues, along with 15 adjuvant chemotherapy regimens, were obtained from Harbin Medical University Cancer Hospital/Center in Harbin, China. All samples were frozen in liquid nitrogen immediately after surgical resection, and only tumors with > 80% tumor cells were selected for RNA extraction. Two independent senior pathologists confirmed the pathological diagnosis and molecular subtype of each cancer tissue. This study conformed to the clinical research guidelines and was approved by the research ethics committee of Harbin Medical University Cancer Hospital. We obtained written informed consent from all patients. For 184 patients, telephone follow-up was performed to verify survival status after primary treatment. Among them, 159 patients received paclitaxel treatment.

### Cell culture and treatments

The human cervical cancer cell line HeLa and 10 breast cancer cell lines (MCF7, T47D, UACC-812, SK-BR-3, MDA-MB-453, MDA-MB-231, Hs578T, HCC70, BT549, and MDA-MB-468) were obtained from the Chinese Academy of Sciences Cell Bank and Cellbio (China), respectively. All cell lines were periodically authenticated (Cellbio). Unless specifically indicated, cells were cultured in DMEM (R10–017-CV, Corning, USA), RPMI-1640 (R10–040-CM, Corning) or Leibovitz’s L15 (PYG0038, Boster, China) medium supplemented with 10% fetal bovine serum (0500, ScienCell, USA) at 37 °C with 5% CO_2_ or air and 95% humidity. In estrogen-related experiments, MCF7 and T47D cells were washed three times with phenol red-free DMEM and subjected to hormone deprivation for up to 3 days with 10% activated charcoal-absorbed fetal calf serum (FCS; P30–2302, PAN) before proceeding to the next steps. The reagents used were 17 β-oestradiol (E8875, Sigma, USA), the estrogen receptor antagonist tamoxifen (T5648, Sigma) and ICI 182780 (Asc-131, Ascent Scientific, USA). Autophagosome and LC3 experiments were performed as previously described [15] and were performed in one of two ways. Cells (10–20 × 10^4^ cells/ml) were plated in medium containing 10% serum. After 24 h, the medium was changed to that containing 0.1% serum, and the cells were collected 48 h later with or without 100 mM CQ (ab142116, Abcam, USA) treatment for 1 h before collection. Alternatively, the cells were cultured with EBSS (E2888, Sigma) for the indicated durations before collection. For the ubiquitination assay, HeLa cells were treated with MG132 (10 μM/l) (ab141003, Abcam) for 40 min. Protein levels were detected by immunoblotting and quantified by densitometry. For the CHX chase assay, HeLa cells were incubated with 50 μg/ml CHX (Catalogue Number HY-12320, MCE, USA) for the indicated durations (0,15, 30, 60, 120, and 180 min) as previously described [16]. For transcriptional inhibition experiments, actinomycin D (6 μg/ml) (A1410, Sigma) was added to the cells, and samples were harvested at the indicated time points (0, 0.5, 1, 2, 4, and 8 h) as previously described [17].

### RNA extraction, reverse transcription and quantitative RT-PCR and PCR array

Total RNA and miRNA were extracted from cells using the E.Z.N.A.® Total RNA Kit I (Catalogue Number R6834–01, Omega Bio-Tek, USA). First-strand cDNA was prepared with the Transcriptor First Strand cDNA Synthesis Kit (Catalogue Number 04897030001, Roche, USA). Real-time PCR was performed using FastStart Universal SYBR Green Master (ROX) (Catalogue Number 04913914001, Roche) on a 7500 Fast Real-Time PCR system (ABI, USA). For quantification of gene expression, we used the 2^-ΔΔCt^ method. *GAPDH* expression was used for normalization. The primer sequences were synthesized by Shanghai Generary Biotech Co., Ltd. and are included in Additional file 1: Table S1.

### Lentivirus production and infection

Recombinant lentiviruses expressing the lncRNA *EGOT*, shEGOT, shITPR1, shESR, *EGOT*-MS2, Flag-MCP2 and controls were constructed by Sangon Biotech Company (China). Concentrated viruses were used to infect 5 × 10^5^ cells in a 6-well plate with 4–6 μg/ml polybrene (107,689, Sigma). The infected cells were then subjected to selection with 1 μg/ml puromycin (Catalogue Number 540411, Calbiochem, USA) for two weeks. Stable overexpression cell lines or knockdown cell lines were identified using qRT-PCR or western blotting. The shRNA sequences are provided in Additional file 1: Table S1.

### Co-immunoprecipitation, western blot assay and antibodies

Co-immunoprecipitation assays were carried out by using the Pierce™ Crosslink Magnetic IP/Co-IP Kit (Catalogue Number 88805, Thermo Fisher, USA) according to the manufacturer’s protocol. Western blotting was performed according to the previously described procedures [18]. Anti-LC3B (ab48394, 1:1000), anti-IP3R (ab5804, 1:1000) antibody, anti-SQSTM1/P62 (ab91526, 1:1000) antibody, anti-hnRNPH1 (ab10374, 1:1000) antibody, anti-HMGB1 (ab79823, 1:1000) antibody, anti-RIP140 (ab42126,1:500) antibody, goat anti-rabbit IgG H&L (HRP) (ab6721, 1:10,000), and goat anti-mouse IgG H&L (HRP) (ab6789, 1:10,000) were obtained from Abcam. Anti-tubulin (sc-73,242, 1:1000) antibody was purchased from Santa Cruz Biotechnology. Anti-estrogen receptor (WL00940, 1:1000) antibody and anti-GAPDH (WL01114, 1:1000) antibody were obtained from Wanleibio (China). Anti-Bax (D2E11, 1:1000) (5023) antibody, anti-Bcl2 (2872, 1:1000) antibody and c-Jun (60A8) Rabbit mAb (#9165, 1:500) antibody were obtained from Cell Signaling Technologies (CST). Total cell lysates were prepared using a 1 × sodium dodecyl sulfate buffer. Identical quantities of proteins were separated by sodium dodecyl sulfate-polyacrylamide gel electrophoresis and transferred onto nitrocellulose filter membranes. After incubation with specific antibodies, the blots were incubated with goat anti-rabbit IgG H&L (HRP) and goat anti-mouse IgG H&L (HRP) for 1 h at room temperature. The proteins were detected using a FluorChem HD2 (Protein Simple, USA).

### Tandem mRFP-GFP fluorescence microscopy

Tandem monomeric RFP-GFP-tagged LC3 (tfLC3) (HB-AP2100001, Hanbio, China) was used to monitor autophagic flux as previously reported. LC3-II relocalized to the autophagosomal membranes during autophagy. Thus, the accumulation of mRFP-GFP-LC3 puncta is an effective way to detect autophagosomes. When tfLC3 is located in autolysosomes, this form of LC3 displays only red fluorescence since the GFP signal is sensitive to the acidic condition in the lysosome lumen, whereas the RFP signal is more stable. To evaluate tandem fluorescent LC3 puncta, 48 h after tfLC3 transfection, cells were washed once with 1 × PBS, incubated with EBSS (E2888, Sigma) for the indicated durations and then directly sent out for confocal microscopy analysis. Images of samples were captured using a Zeiss LSM 710 confocal microscope system (Carl Zeiss, Germany) and processed with ZEN LE software (Carl Zeiss).

### Subcellular fractionation

Nuclear/cytoplasmic isolation was carried out by using the NE-PER™ Nuclear and Cytoplasmic Extraction Reagents (Catalogue Number 78835, Thermo Fisher) according to the manufacturer’s protocol. Subcellular fractions were prepared as follows. Cytoplasmic and nuclear fractions were divided for RNA extraction. *GAPDH* and *U1* were used as qRT-PCR markers of cytoplasmic and nuclear RNAs, respectively.

### Transmission electron microscopy

Conventional electron microscopy was performed as previously described [19]. In brief, cells were fixed with 2.5% glutaraldehyde and then postfixed with 1% osmium tetroxide, dehydrated in a graded ethanol series and embedded in Embed 812 resin. Ultrathin sections were mounted on copper grids and then double-stained with uranyl acetate and lead citrate. The samples were examined and photographed with an FEI Tecnai spirit transmission electron microscope.

### RNA-FISH assay

To detect *EGOT* and *pre-ITPR1* mRNAs, we used the QuantiGene® ViewRNA ISH Cell Assay Kit (Catalogue Number QVC0001, Thermo Fisher) to perform the QuantiGene ViewRNA FISH assay according to the manufacturer’s protocol. *EGOT*, *ITPR1* and *ACTB* (as control) hybridization was carried out using cy3, cy5, and 488-nm DNA-oligonucleotide probes in a moist chamber, respectively. After digestion with a working protease solution, slides were incubated with RNase III (AM2290, Life Technologies, USA) or RNase A (AM2272, Life Technologies) for 2 h if RNase enzymatic activity was to be determined. Standard immunofluorescence and imaging were performed by confocal microscopy. The details of the probe sets and corresponding gene sequences are provided in Additional file 1: Table S2.

### TUNEL assay

To detect apoptosis in sections of tumor tissues, TUNEL assay was performed according to the manufacturer’s instructions (Catalogue Number 11684795910, Roche) as previously described [20]. The sections were analyzed by fluorescence microscopy (Olympus, Japan).

### RNA pull-down assay

The Flag-MS2bp-MS2bs-based RNA pull-down assay was carried out by using the Anti-Flag M2 Affinity Gel (Catalogue Number A2220, Sigma) as previously described [21]. In short, lentivirus Flag-MS2bp and lentivirus EGOT MS2bs were cotransfected into breast cancer cells, and the cells were harvested after 48 h. Approximately 1 × 10^7^ cells were lysed in soft lysis buffer (20 mM Tris-Cl, pH 8.0, 10 mM NaCl, 1 mM EDTA, and 0.5% NP-40) containing RNasin (80 units/ml). Fifty microliters of Anti-Flag M2 Magnetic beads was added to each binding reaction tube and incubated at 4 °C overnight. The beads were washed three times with lysis buffer and boiled in 1x loading buffer for 10 min. Finally, the retrieved proteins were analyzed by a NanoLC-ESI-MS/MS system (ProtTech, China).

### Chromatin immunoprecipitation (ChIP) and ChIP-seq

Analysis of genome-wide NRIP1 and AP-1 occupancy was carried out using specific and internally validated antibodies. We purchased the EZ-ChIP™ Chromatin Immunoprecipitation Kit (Catalogue Number #17–371, Millipore, USA) to perform ChIP and ChIP-seq using MCF7 cells as previously described [22]. Briefly, MCF7 cells were subjected to hormone deprivation for up to 3 days and then treated with 1 nM E2 or ethanol (control) for 6 h. Approximately 2 × 10^7^ cells were used for each ChIP or ChIP-Seq assay. Chromatin DNA precipitated by polyclonal antibodies against AP-1 or NRIP1 was purified with the Universal DNA purification kit (DP214, Tiangen, China) according to the manufacturer’s protocol. Rabbit anti-RIP140 (ab42126, Abcam) and rabbit anti-c-JUN (60A8) (Catalogue Number #9165, CST) antibodies and rabbit IgG (sc-2027, Santa Cruz, USA) were used. ChIP-PCR enrichment of target loci was normalized to input DNA and reported as % input ± s.e.m. ChIP libraries were prepared using ChIP DNA according to the BGISEQ-500ChIP-Seq library preparation protocol. In-depth whole-genome DNA sequencing was performed by BGI (Shenzhen, China). ChIP-seq data were deposited in the NCBI SRA: SRP149488 (https://www.ncbi.nlm.nih.gov/sra/SRP149488).

### Transient transfection of cells

For downregulation of NRIP1 and hnRNPH1 expression, siRNAs targeting NRIP1 and hnRNPH1 were purchased from Sigma. Cancer cells were transfected with 100 pM siRNA using Lipofectamine 2000 (11668–019, Invitrogen, USA). The siRNA sequences are provided in Additional file 1: Table S1. After HeLa and T47D cells (5 × 10^5^ cells/well) were cultured in 6-well plates for 24 h, siRNA or the corresponding controls were transfected into the cells by Lipofectamine 2000 reagent in accordance with the manufacturer’s protocol. Total RNA was extracted 48 h after transfection.

### Animal experiment

Female athymic BALB/c nude mice (5 weeks old) were obtained from Beijing Vital River Laboratory Animal Technology Co, Ltd. (Beijing, China). All of the animal experiments were performed according to approved protocols and in accordance with the guidelines of the Guide for the Care and Use of Laboratory Animals (Institute of Laboratory Animal Resources, Commission on Life Sciences, National Research Council). The protocol was approved by the Institutional Animal Care and Use Committee of Center of Harbin Medical University. Approximately 8 × 10^6^ UACC-812 cells resuspended in 0.2 ml of 25% phenol red-free Matrigel (Catalogue Number 356234, Corning) with 0.9% NaCl were injected in the axilla of five-week-old BALB/c mice. After the tumors grew to approximately 300–500 mm^3^ in size, 15 mg/kg paclitaxel was administered once every 4 days for a total of three courses. Tumor volume was measured once every 2 days by using calipers at the indicated time points. The tumor volume was estimated by the following formula: length × width × width/2. The whole body weight of mice was measured once every 2 days as indicated. All mice were euthanized by intraperitoneal injection of 200 mg/kg pentobarbital at the end of the experiment.

### Immunohistochemistry

Immunohistochemical (IHC) detection of ITPR1 and LC3B was performed on each slide. Each section was incubated with anti-LC3B (1:200) antibody or anti-IP3R (1:200) antibody solution. The proportion and intensity of ITPR1 and LC3 staining were evaluated in a series of 10 randomly selected high-power fields (magnification, 400**×**), which were considered to represent the average expression. The IHC staining intensity was graded as 0 (no staining), 1 (weak staining = light yellow), 2 (moderate staining = yellow brown) or 3 (strong staining = brown). The proportion of positively stained tumor cells in a field was scored as 0 (no positive tumor cells), 1 (fewer than 10% positive tumor cells), 2 (10–50% positive tumor cells) or 3 (more than 50% positive tumor cells). The staining index (SI) for each sample was obtained by multiplying the intensity and proportion values, with a score of less than 4 being classified as low expression.

### Library preparation for lncRNA sequencing

A total of 3 μg of RNA per sample was used for downstream RNA sample preparation. Ribosomal RNA was removed using the Ribo-Zero™ Gold kit (Epicentre, Wisconsin, USA). Subsequently, sequencing libraries were generated according to the manufacturer’s recommendations, and the libraries were sequenced on an Illumina HiSeq 2500 platform to generate 100-bp paired-end reads. Raw sequencing and processed RNASeq data from this study have been deposited into the NCBI GEO database under accession number GSE71651 (http://www.ncbi.nlm.nih.gov/geo/query/acc.cgi?token=obcxosaur xoppwx & acc = GSE71651).

### Access and analysis of public data

GDS723, GDS4121, GDS1453 and GSE11324 were downloaded from GEO datasets (http://www.ncbi.nlm.nih.gov/geo/) and processed according to our previous study [23]. Genome-wide *EGOT* and *ITPR1* expression profiles and clinical pathology information for human cancers were downloaded from The Cancer Genome Atlas (TCGA) (https://tcga-data.nci.nih.gov/), International Cancer Genome Consortium (ICGC) (http://icgc.org/) and Cancer Cell Line Encyclopedia (CCLE) (http://www.broadinstitute.org/ccle). All transcripts were normalized by log_2_ transformation. The expression of *EGOT* or *ITPR1* was dichotomized using a study-specific median expression as the cut-off to define high values (at or above the median) versus low values (below the median). Guilt-by-association analysis was performed according to our previous study [23]. Correlations between genes were assessed by Pearson correlation coefficients. Unpaired Student’s t-tests were used to detect significant differences among tumors or between tumor and normal samples. The overall survival (OS) and relapse-free survival (RFS) were calculated as the time from surgery until the occurrence of death and relapse, respectively. The log-rank test was used to examine the survival difference between different patient groups. All statistical tests were two-sided, and *P* < 0.05 indicated statistical significance.

### Statistical analysis

Data are presented as the mean ± s.d. of at least three independent experiments for each cellular experimental group and at least five independent experiments for each animal group. Student’s t-tests were used to determine statistically significant differences between groups. Correlations between *EGOT* expression and a pathological response were determined by the chi-square test. All statistical tests were two-sided, and *P* < 0.05 indicated statistical significance. Statistical analysis was performed using R.3.4 graphics software and GraphPad Prism software (GraphPad Software, USA).

## Results

### Ai-lncRNA *EGOT* enhances paclitaxel sensitivity in human cancer

**Fig. 1:**
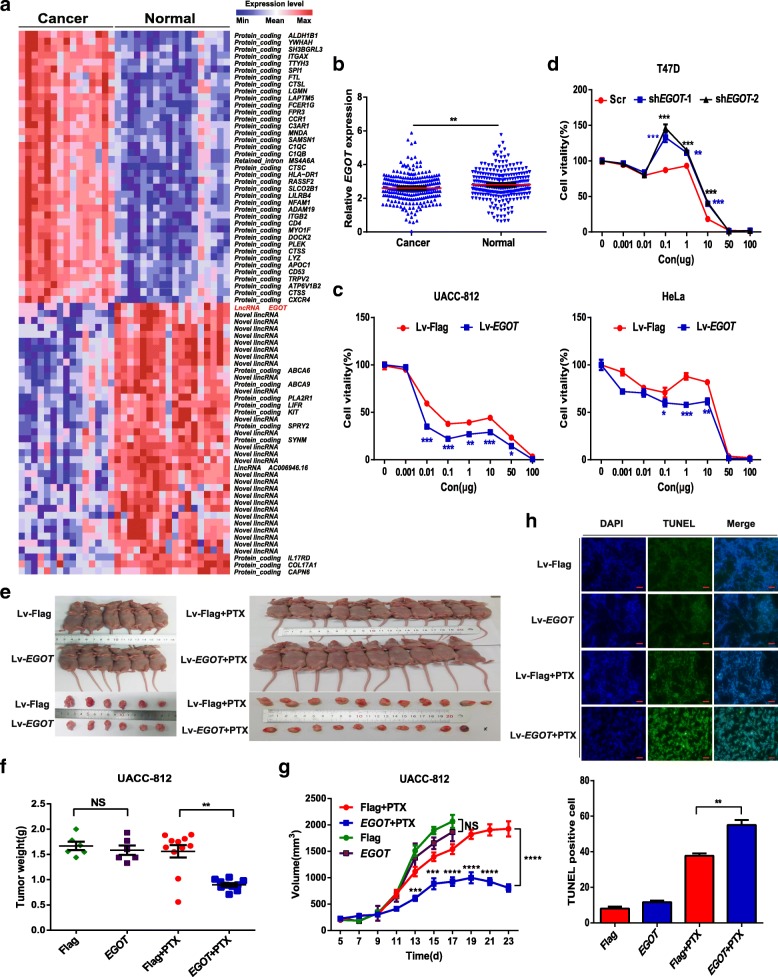
Ai-lncRNA *EGOT* enhances paclitaxel sensitivity in human cancer. **a** Heatmap of the whole transcriptomes of 33 breast specimens indicating that lncRNA *EGOT* expression levels are lower in cancer tissues than in normal tissues. Colors correspond to the expression level indicated by the log_2_-transformed scale bar below the matrix. Red and blue reflect Max and Min levels, respectively. **b** qRT-PCR analysis of *EGOT* expression in breast cancer tissues and adjacent normal tissues from 258 patients in the HMUCC cohort. *GAPDH* served as a reference for *EGOT*. **c**, **d** Cell viability was analyzed by CCK-8 assay after paclitaxel treatment for 48 h in *EGOT* overexpression and knockdown cells. **e** Mice and tumours from the UACC-812 Lv-*EGOT* and control (Lv-Flag) groups with or without treatment of paclitaxel. **f** The weight of tumours excised from mice in the UACC-812 Lv-*EGOT* and control groups with or without treatment of paclitaxel. **g** The volumes of tumours established in UACC-812 Lv-*EGOT* and control groups with or without 15 mg/kg paclitaxel once every four days at the indicated time (day 11, day 15 and day 19). **h** Representative sections (upper) and average number of TUNEL-positive cells (bottom) in subcutaneous tumors. Scale bar, 50 μm. Data are shown as the mean ± s.d. Student’s t-test was used for statistical analysis: * *P* < 0.05; ** *P* < 0.01; *** *P* < 0.001; and **** *P* < 0.0001. Data represent at least three independent experiments

Whole transcriptome sequencing of 33 breast specimens showed that Ai-lncRNA *EGOT* was expressed at low levels in cancer tissues compared with its levels in adjacent normal tissues (Fig. 1a and Additional file 2: Figure S1a). This result was subsequently validated in 258 pairs of breast cancer tissues and adjacent normal tissues in the Harbin Medical University Cancer Centre (HMUCC) cohort (Fig. 1b) and in data from other cancer contexts in TCGA (Additional file 2: Figure S1b).

To further investigate the biological functions of *EGOT*, we performed guilt-by-association analysis and found that *EGOT* was involved in multiple cellular microtubule-associated functions (Additional file 1: Table S3). Because paclitaxel is commonly used as a microtubule-disrupting agent, we suspected that *EGOT* may impact paclitaxel sensitivity in tumor cells. As expected, analysis of public data from CCLE showed that *EGOT* increases the sensitivity of tumor cells to paclitaxel (Additional file 2: Figure S1c). To validate the potential function of *EGOT* in mediating the paclitaxel response, we then generated stable overexpression and knockdown of *EGOT* in cancer cell lines (Additional file 2: Figure S1d-f). As shown in Fig. 1c and Additional file 2: Figure S1g, we determined that *EGOT* overexpression significantly increased the sensitivity of cancer cells to paclitaxel. Conversely, silencing *EGOT* protected cancer cells from paclitaxel (Fig. 1d).

Furthermore, we found that there was no distinct difference of xenografts in the presence of *EGOT* or control without treatment of paclitaxel in UACC-812 cell xenograft models (Fig. 1e-g). However, *EGOT* overexpression (Additional file 2: Figure S1 h) in UACC-812 and HeLa cell xenografts grew more slowly than control ones when treated with paclitaxel (Fig. 1e and Additional file 2: Figure S1i). Moreover, the weight and volume of the tumors were decreased in the *EGOT* overexpression group compared with those in the control group with treatment of paclitaxel (Fig. 1f and g). Similar results were obtained in HeLa cell xenografts (Additional file 2: Figure S1j and k). More importantly, cell apoptosis was much higher in the *EGOT* overexpression group than in the control group when treated with paclitaxel (Fig. 1h). Collectively, these data demonstrate that *EGOT* enhances the sensitivity of human cancer cells to paclitaxel both in vitro and in vivo.

### *EGOT* expression is positively correlated with ITPR1 expression

**Fig. 2:**
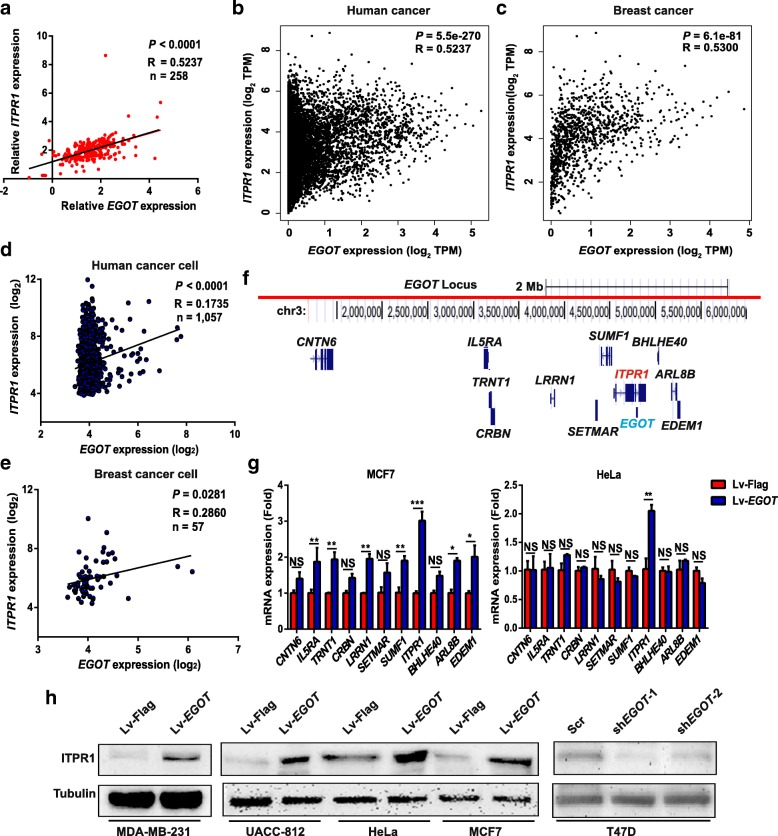
*EGOT* expression is positively related to ITPR1 expression. **a** Correlation between *EGOT* and ITPR1 expression in 258 breast cancer tissues in the HMUCC cohort. *EGOT* and ITPR1 levels (normalized to *GAPDH*) were subjected to Pearson’s correlation analysis. **b**, **c** Correlation between *EGOT* and *ITPR1* expression in 33 cancer types (*n* = 9664) (**b**) and in breast cancer (*n* = 1085) (**c**). Data were downloaded from the TCGA portal, and the expression levels of *EGOT* and *ITPR1* are indicated by TPM values (log_2_). **d**, **e** Correlation between *EGOT* and *ITPR1* expression in 1057 human cancer cell lines (**d**) and 57 breast cancer cell lines (**e**). Data were downloaded from the CCLE. *EGOT* and *ITPR1* levels (normalized to *GAPDH*) were subjected to Pearson’s correlation analysis. **f** Schematic illustration of genes near the *EGOT* locus (less than 2 Mb). Location information was obtained from the UCSC Genome Browser (http://genome.ucsc.edu/cgi-bin/hgGateway). **g** Expression of nearby genes in MCF7 *EGOT* overexpression cells (left) and HeLa *EGOT* overexpression cells (right). Data are shown as the means ± s.d. Student’s t-test was applied for statistical analysis: * *P* < 0.05; ** *P* < 0.01; and *** *P* < 0.001. **h** Protein expression levels of ITPR1 in *EGOT* overexpression and knockdown cells. Data represent at least three independent experiments

We then sought to identify interacting partner(s) involved in *EGOT*-mediated paclitaxel sensitivity. Certain mammalian lncRNAs are embedded in the intronic-antisense regions of protein-coding genes and regulate the genes to exert their function [24]. Thus, we hypothesized the existence of a functional relationship between *EGOT* and *ITPR1*. To study this possibility, by analyzing whole-transcriptome sequencing data, we found that *EGOT* expression was positively correlated with *ITPR1* expression at the mRNA level (Additional file 2: Figure S2a). The result was validated using breast cancer specimens in the HMUCC cohort (Fig. 2a); moreover, *EGOT* expression was also positively correlated with ITPR1 protein expression in the HMUCC cohort (Additional file 2: Figure S2b). To expand the generalizability of our results, human cancer data from 33 cancer contexts, including breast cancer, in TCGA were analyzed, and the results further validated as above (Fig. 2b and c; Additional file 1: Table S4). Furthermore, examination of CCLE data also revealed that *EGOT* expression was positively correlated with *ITPR1* mRNA expression in human breast cancer cell lines and other cancer cell lines (Fig. 2d and e; Additional file 2: Figure S2c-f). These results indicate that *EGOT* expression is positively correlated with ITPR1 expression in cancer.

To further validate the relationship between *EGOT* and ITPR1expression, we detected the genes near the *EGOT* locus (less than 2 Mb). *EGOT* overexpression was shown to increase the expression of *ITPR1* mRNA (Fig. 2f and g), while *EGOT* knockdown decreased the expression of *ITPR1* mRNA (Additional file 2: Figure S2 g). Consistent with these results, ITPR1 protein levels were also increased following *EGOT* overexpression and were decreased following *EGOT* knockdown (Fig. 2h). However, *EGOT* expression did not significantly change following ITPR1 knockdown (Additional file 2: Figure S2 h). Collectively, these data suggest that *EGOT* may induce ITPR1 expression in human cancer.

### *EGOT* induces autophagy to enhance paclitaxel sensitivity through ITPR1

To illustrate that ITPR1 plays a functional role in autophagy in human cancer cells, we investigated autophagic activity in cells after 48 h of growth under low-serum (0.1%) conditions or in Earle’s balanced salt solution (EBSS) to simulate stress-induced autophagy, as previously described [15]. Knockdown of ITPR1 decreased LC3-II/LC3-I levels and increased P62 expression in the presence of chloroquine (CQ) or EBSS (Additional file 2: Figure S3a and b). Moreover, nutrient starvation failed to induce autophagy when ITPR1 was knocked down in cancer cells, as shown by confocal microscopy examination 48 h after mRFP-GFP-LC3 adenovirus vector transfection (Additional file 2: Figure S3c and d). Additionally, electron microscopy examination indicated that ITPR1 knockdown reduced the number of autophagic vesicles (Additional file 2: Figure S3e).

**Fig. 3:**
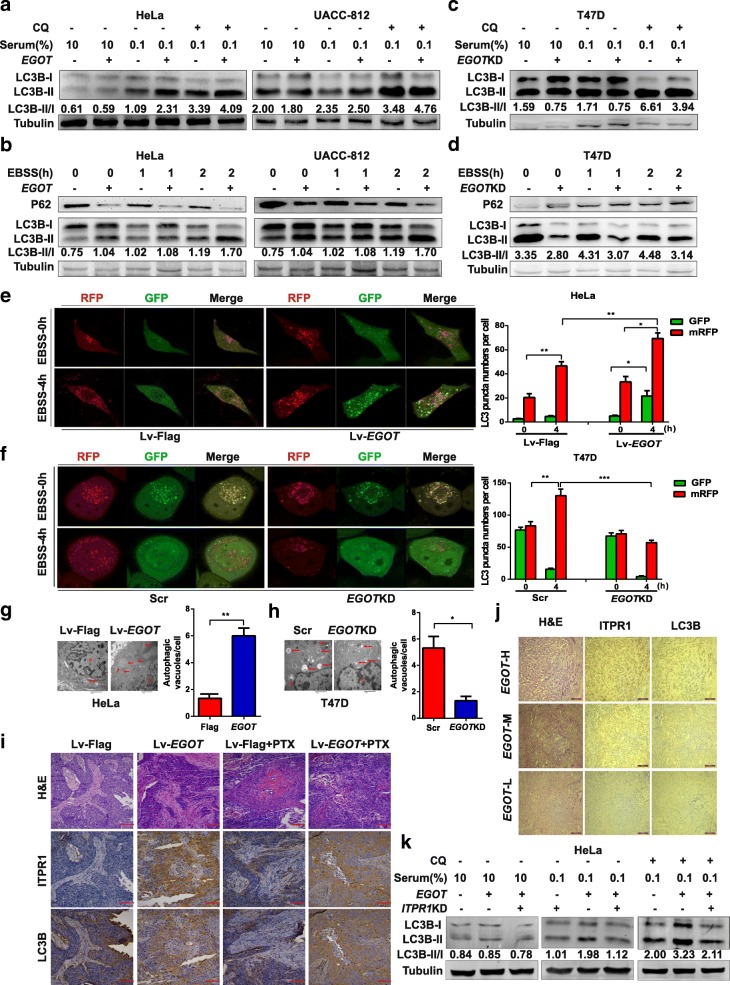
*EGOT* induces autophagy to enhance paclitaxel sensitivity through ITPR1. **a** Western blot showing the effects of *EGOT* overexpression on LC3-II/LC3-I levels in HeLa (left) and UACC-812 cells (right) treated with CQ. **b** Western blot showing the effects of *EGOT* overexpression on LC3-II/LC3-I levels and P62 expression in HeLa (left) and UACC-812 cells (right) treated with EBSS. **c** Western blot showing the effects of *EGOT* overexpression on LC3-II/LC3-I levels in T47D cells treated with CQ. **d** Western blot showing the effects of *EGOT* overexpression on LC3-II/LC3-I levels and P62 expression in HeLa (left) and UACC-812 cells (right) treated with EBSS. **e**, **f** Confocal microscopy showing the effects of EBSS incubation on mRFP-GFP-LC3 distribution in HeLa *EGOT* overexpression cells (**e**) or T47D *EGOT* knockdown cells (**f**) 48 h after mRFP-GFP-LC3 adenovirus transfection (10,000× magnification). **g**, **h** Representative electron microscopy images and quantification of autophagic vacuoles in *EGOT* overexpression cells (**g**) and EGOT knockdown cells (**h**). Scale bar, 2 μm. Arrows depict autophagosomes, and the nucleus is denoted by N. **i** Representative hematoxylin and eosin (H&E), ITPR1, and LC3B staining of orthotopic xenograft sections from UACC-812 *EGOT* overexpression and control groups treated with or without paclitaxel. Scale bar, 400 μm. **j** Representative H&E, ITPR1 and LC3B staining in sections from 258 breast cancer tissues in HMUCC cohort. Scale bar, 200 μm. *EGOT*-H, *EGOT*-M, and *EGOT*-L represent high, medium, and low expression of *EGOT*, respectively. **k** Western blot showing LC3-II/LC3I levels in HeLa cells treated with CQ, different serum condition, *EGOT* overexpression or *ITPR1* knockdown. Data are shown as the mean ± s.d. Student’s t-test was used for statistical analysis: * *P* < 0.05; ** *P* < 0.01; *** *P* < 0.001; and **** *P* < 0.0001. Data represent at least three independent experiments

We next explored whether *EGOT* was involved in autophagy via ITPR1 in the presence of CQ or EBSS. As expected, overexpression of *EGOT* increased LC3-II/LC3-I levels and reduced P62 expression in HeLa and breast cancer cells (Fig. 3a and b; Additional file 2: Figure S3f and g), while knockdown of *EGOT* reduced LC3-II/LC3-I levels and increased P62 expression (Fig. 3c and d). Meanwhile, overexpression of *EGOT* induced autophagy, as shown by the increases in mRFP-GFP-LC3 puncta accumulation and the number of autophagic vesicles (Fig. 3e and g; Additional file 2: Figure S3 h). In contrast, knockdown of *EGOT* caused the opposite changes (Fig. 3f and h). Moreover, *EGOT*-induced autophagy could be reversed by ITPR1 knockdown in HeLa and MCF7 cells (Fig. 3k; Additional file 2: Figure S3i). These results revealed that *EGOT* can at least partly convey the autophagic signal via ITPR1.

Furthermore, by analyzing CCLE data, we found that *ITPR1* may impact paclitaxel sensitivity in tumor cells (Additional file 2: Figure S3j). The result was validated by experimental evidence showing that ITPR1 knockdown conferred protection to cancer cells from paclitaxel (Additional file 2: Figure S3k). Moreover, paclitaxel sensitivity induced by *EGOT* overexpression could be repressed by ITPR1 knockdown in vitro (Additional file 2: Figure S3 l). In addition, *EGOT*-overexpressing tumors exposed to paclitaxel treatment in vivo expressed high levels of ITPR1 and LC3B proteins (Fig. 3i; Additional file 2: Figure S3 m and n). Consistently, breast cancer tissues with higher *EGOT* levels showed higher ITPR1 and LC3B levels in the HMUCC cohort, suggesting that the increased expression of *EGOT* may force a connection with the autophagic pathway via ITPR1 (Fig. 3j). These results indicate that *EGOT* enhances paclitaxel sensitivity through ITPR1 in human cancer.

### *EGOT* regulates ITPR1 expression both *in cis* and *in trans* in human cancer

Emerging evidence shows that lncRNAs can regulate gene expression and protein functions in *trans* or *in cis* [9]. Some lncRNAs are known to be involved in regulating protein synthesis or protein stability in mammalian cells [25]. We found that neither ITPR1 protein synthesis nor protein stability appeared to have any significant effect on the overexpression of *EGOT* via assays using the protein synthesis inhibitor cycloheximide (CHX) and the proteasome inhibitor MG132 (Additional file 2: Figure S4a and b).

**Fig. 4:**
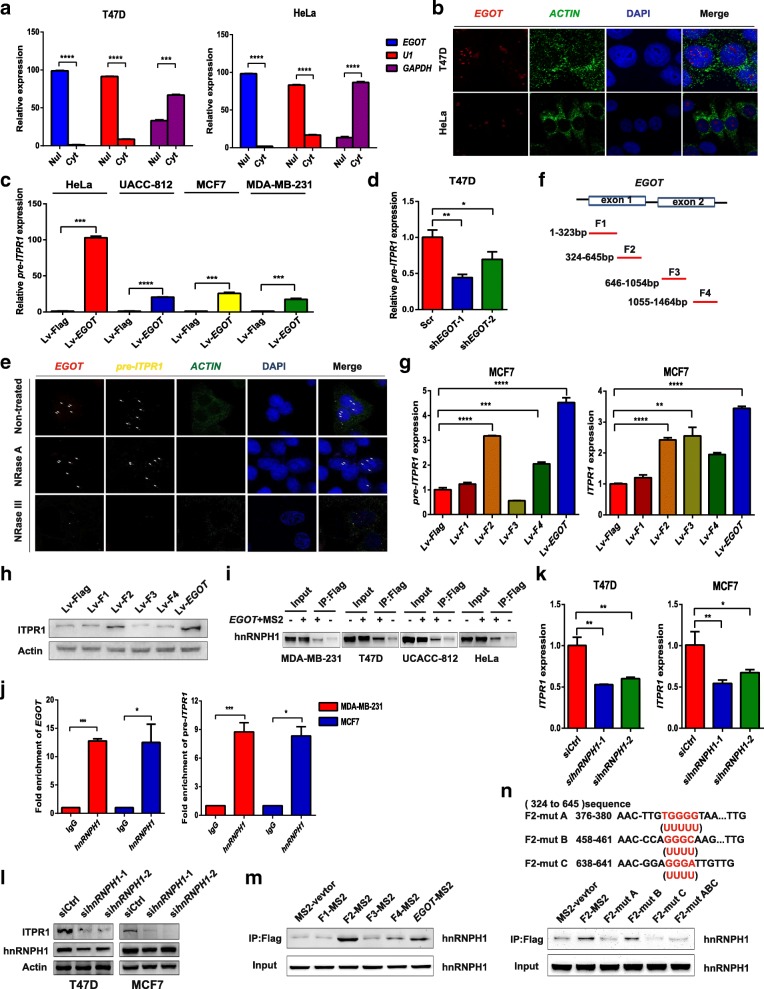
*EGOT* regulates ITPR1 expression *in cis* and *in trans* in human cancer. **a** Nuclear and cytoplasmic fractions of HeLa and T47D cells were subjected to qRT-PCR. U1 is the nuclear (Nul) positive control; GAPDH is the cytoplasmic (Cyt) positive control. **b** RNA-FISH performed in HeLa and T47D cells. *EGOT* probes are red; *ACTIN* probes are green; and *ACTIN* served as the positive control (1000 × magnification). **c**, **d** Expression of pre-ITPR1 in *EGOT* overexpression and knockdown cells by qRT-PCR. **e** Combined RNase resistance and dual-RNA-FISH analysis. Before hybridization, T47D cells were treated with RNase A or RNase III. Hybridization was performed with specific probes against *EGOT* and *ITPR1* transcripts. Nuclei are stained with DAPI. *EGOT* probes are red; *ITPR1* probes are yellow; and *ACTIN* is the positive control. Arrows indicate foci (1000 × magnification). **f** Domain mapping of the *EGOT* transcript. **g**, **h** Expression of *pre-ITPR1* and *ITPR1* mRNA (**g**) and protein (**h**) in MCF7 cells transfected with different fragments of *EGOT* lentivirus. **i** An RNA pull-down assay was conducted using the Flag-MS2bp-MS2bs-based system followed by western blotting of lysates from MDA-MB-231, T47D, and UACC-812 cells after transfection with MS2-*EGOT* and MS2-vector (control). **j** Antibodies against hnRNPH1 were used for RIP, followed by qRT-PCR, in MCF7 and MDA-MB-231 cells. **k, l** ITPR1 mRNA expression was analyzed by qRT-PCR after hnRNPH1 knockdown via siRNAs (**k**), followed by western blotting (**l**). **m** The RNA pull-down assay was conducted using the Flag-MS2bp-MS2bs-based system in T47D cells after transfection with MS2-*EGOT* lentivirus along with different fragments of *EGOT* lentivirus, followed by western blotting. **n** Western blot of hnRNPH1 pulled down by F2-MS2 and mutated F2 RNA in 293 T cells. The red underlined sequences indicate the potential binding sites that were mutated into Us in F2, while the A, B and C binding sites were mutated at the same time to F2-mut ABC. Data are shown as the mean ± s.d. Student’s t-test was used for statistical analysis: * *P* < 0.05; ** *P* < 0.01; *** *P* < 0.001; and **** *P* < 0.0001. Data represent at least three independent experiments

By fractionated nuclear and cytoplasmic RNA analysis and RNA fluorescence in situ hybridization (RNA-FISH) examination in four cancer cell lines, we found that *EGOT* is mainly expressed in the nucleus (Fig. 4a and b; Additional file 2: Figure S4c and d), suggesting that *EGOT* exerts its regulatory function for ITPR1 at the transcriptional level. Of note, ectopic expression of *EGOT* induced endogenous upregulation of *pre-ITPR1* (Fig. 4c), while *EGOT* knockdown suppressed endogenous expression of *pre-ITPR1* (Fig. 4d). Moreover, the stability of *pre-ITPR1* mRNA was increased in *EGOT*-overexpressing cancer cells compared with that in control cells, as shown in the transcriptional inhibition experiments (Additional file 2: Figure S4e).

Given that *EGOT* is complementary to intronic sequences of the *ITPR1* and is transcribed in the antisense direction, we hypothesized that a double-stranded (ds) RNA forms between *EGOT* and *pre-ITPR1*. As expected, dual-RNA-FISH assay indicated that *EGOT* and *pre-ITPR1* hybridized in the same nuclear foci (Fig. 4e). These foci were completely depleted upon treatment with RNase III, which degrades only dsRNA, but not with RNase A, which degrades only single-stranded (ss) RNA (Fig. 4e), thereby indicating the formation of dsRNA from the physical association of *EGOT* and *pre-ITPR1* (Additional file 2: Figure S4f). Furthermore, through domain mapping, two exons of *EGOT* were divided into four fragments (Fig. 4f). Interestingly, *EGOT* fragment 2 (324–645 nucleotides) in exon 1 increases ITPR1 protein levels as well as *ITPR1* and *pre-ITPR1* mRNA levels, whereas other fragments do not possess these capacities (Fig. 4g and h). Taken together, these data indicate the pivotal roles of *EGOT* fragment 2 (324–645 nucleotides) in exon 1, which binds to *pre-ITPR1* to control ITPR1 expression *in cis* in human cancer.

Accumulating evidence suggests that lncRNA activity is centered on its ability to bind and regulate epigenetic complexes of methylation, acetylation and phosphorylation [26, 27]. Therefore, by using the Flag-MS2bp-MS2bs-based system (Additional file 2: Figure S4 g) and, subsequently, mass spectrometry examination (Additional file 1: Table S5), hnRNPH1 was pulled down in this process, and the result was validated in four other cancer cell lines (Fig. 4i). RNA immunoprecipitation (RIP) further revealed that hnRNPH1 bound to both *EGOT* and *pre-ITPR1* mRNA (Fig. 4j). Given the earlier evidence that hnRNPH1 interacts with RNA-binding proteins to improve alternative splicing [28], inhibition of hnRNPH1 expression may suppress alternative splicing of its target. As expected, knockdown of hnRNPH1 decreased *ITPR1* mRNA and protein levels (Fig. 4k and l). As *EGOT* fragment 2 (324–645 nucleotides) in exon 1 binds to *pre-ITPR1* mRNA *in cis* (Fig. 4g), ectopic expression of *EGOT* fragment 2 (324–645) resulted in enrichment of hnRNPH1 by the RNA pull-down assays (Fig. 4m). hnRNPH1 has been reported to regulate alternative splicing by binding to specific motifs (GGGA) [29]. Moreover, mutation of the specific binding motifs (mut-A and mut-C) in *EGOT* fragment 2 led to the failure of hnRNPH1 enrichment (Fig. 4n). Altogether, these data demonstrated that hnRNPH1 is recruited by *EGOT* fragment 2 to enhance the alternative splicing of *pre-ITPR1 in trans*.

### *EGOT* expression is transcriptionally regulated by stress in human cancer

Through guilt-by-association analysis, we found that *EGOT* is involved in stress-associated conditions (Additional file 1: Table S1). Stress, including hypoxia, estrogen depletion, radiotherapy, and chemotherapy, has been shown to affect autophagy induction [30–33]. Thus, we first aimed to determine whether *EGOT* is regulated by hypoxia. Hypoxia (1% oxygen) treatment induced a sustained upregulation of *EGOT* in a time-dependent manner in several cell types (Additional file 2: Figure S5a-e). Moreover, by analyzing previously published GEO datasets (GDS4121, GDS1453 and GDS723) involving different stress treatments, we found that *EGOT* was also upregulated in the absence of HGF stimulation or treatment with camptothecin or radiation (Additional file 2: Figure S5f-h). Furthermore, *EGOT* was upregulated in breast cancer patients who underwent neoadjuvant chemotherapy compared with its expression in patients who did not receive such therapy in the HMUCC cohort (Additional file 2: Figure S5i).

**Fig. 5:**
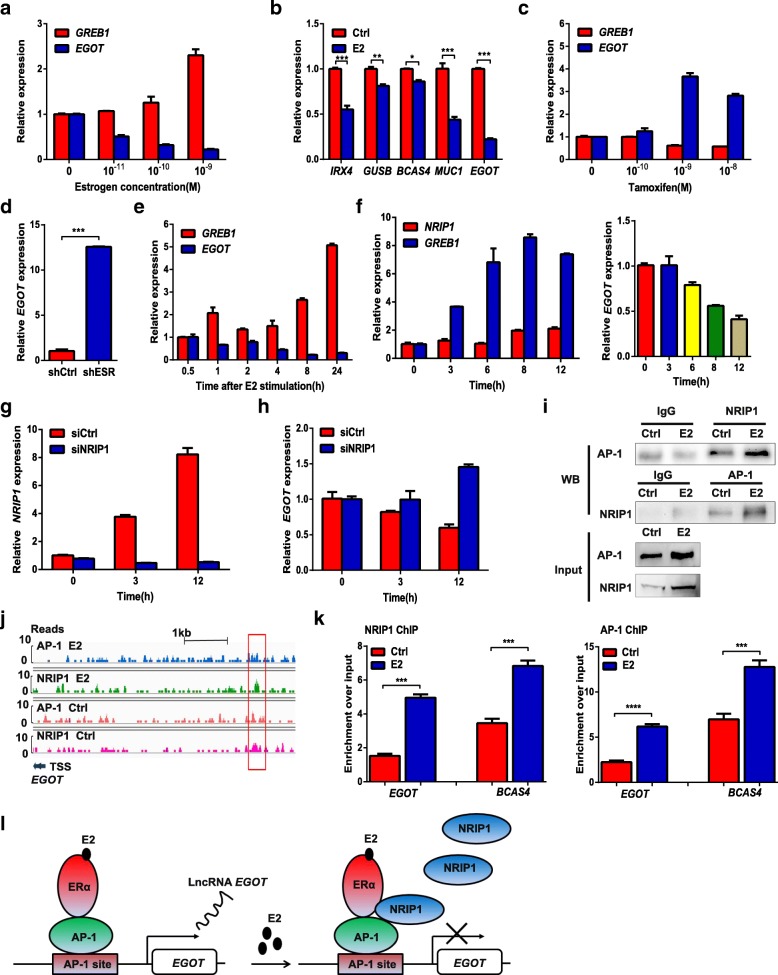
*EGOT* expression is directly repressed by the NRIP1/AP-1 complex. **a** *EGOT* expression determined by qRT-PCR (normalized to *GAPDH*) in MCF7 cells treated with E2. **b** Expression of the estrogen-repressed genes, *IRX4*, *GUSB*, *BCAS4*, *MUC1* and *EGOT*, determined by qRT-PCR in MCF7 cells treated with 1 nM E2 or ethanol (Ctrl) for 6 h. **c** *EGOT* expression determined by qRT-PCR in MCF7 cells treated with different doses of tamoxifen. **d** *EGOT* expression determined by qRT-PCR in MCF7 cells following ESR knockdown. **e** *EGOT* and *GREB1* expression in MCF7 cells treated with 1 nM E2 stimulation at different time points. **f** NRIP1 mRNA and *EGOT* expression in MCF7 cells treated with 1 nM E2 stimulation at different time points. **g**, **h** Control siRNA (siCtrl) or siRNA against NRIP1 (siNRIP1) was transfected into MCF7 cells for 48 h, followed by 1 nM E2 stimulation. NRIP1 mRNA, *GREB1*, and *EGOT* levels were assessed after estrogen stimulation in the presence of siCtrl or siNRIP1. **i** Co-IP assays were conducted using anti-NRIP1 or anti-AP-1 antibodies for endogenous proteins in MCF7 cells treated with or without E2 stimulation. **j** Genome browser view of AP-1 and NRIP1 binding at the promoter of the *EGOT* locus. AP-1 and NRIP1 ChIP-seq was performed in MCF7 cells stimulated with ethanol (Ctrl) or E2. **k** ChIP-PCR showed the binding of AP-1 and NRIP1 to the *EGOT* promoter. The BCAS4 gene was utilized as a positive control. **l** Schematic illustration of NRIP1/AP-1 complex-mediated transcription suppression of *EGOT* expression. MCF7 cells were hormone starved for 3 days and further treated. In A, C, E, and F, *GREB1* was used as an estrogen-inducible positive control gene. In B and D, data are shown as the mean ± s.d. Student’s t-test was used for statistical analysis: * *P* < 0.05; ** *P* < 0.01; and *** *P* < 0.001. Data represent at least three independent experiments

Estrogen (E2) is strongly related to breast cancer and has been shown to inhibit autophagy in a previous report [34]. Regarding the potential role of E2 in regulating *EGOT* expression, E2 indeed suppressed *EGOT* expression in a dose-dependent manner (Fig. 5a). Moreover, analysis from the GSE11324 dataset further certified the relationship of *EGOT* inhibition by E2, which was accordance with our results above (Additional file 2: Figure S6a). Concordantly, the expression levels of several positive control genes that are negatively regulated by E2, such as *IRX4*, *GUSB*, *BCAS4* and *MUC1*, were decreased after E2 stimulation (Fig. 5b). In contrast, *EGOT* expression was induced in response to treatment with the anti-estrogen agents tamoxifen and fulvestrant (ICI) (Fig. 5c and Additional file 2: Figure S6b). In addition, knockdown of the estrogen receptor (ER) in MCF7 cells was shown to induce the expression of *EGOT* by > 10-fold (Fig. 5d), while the expression of the positive control gene *GREB1,* which was reported to be positively regulated by E2, was decreased, and that of the *IRX4,* which was reported to be negatively regulated by E2*,* was increased (Additional file 2: Figure S6c). Furthermore, time course analysis demonstrated that E2 suppressed *EGOT* expression in a time-dependent manner (Fig. 5e). Moreover, dose-dependent inhibition of *EGOT* expression by E2 was also observed in other ER-positive T47D breast cancer cells (Additional file 2: Figure S6d). In all, these results demonstrate that *EGOT* is transcriptionally inhibited by E2 in ER-positive breast cancer cells.

Next, we determined the molecular mechanism underlying such transcriptional repression. Estrogen-inducible NRIP1 acts as a nuclear receptor corepressor and interacts with AP-1 to directly repress target genes via ERE or AP-1 elements [35]. The expression of *NRIP1* was induced, whereas *EGOT* expression was decreased after E2 treatment in MCF7 cells (Fig. 5f). As expected, transcriptional repression of *EGOT* by E2 was markedly reversed and the positive control gene *GREB1* was upregulated in the presence of siNRIP1 (Fig. 5g and h; Additional file 2: Figure S6e), demonstrating that NRIP1 is necessary and involved in this process. Furthermore, coimmunoprecipitation assays revealed that NRIP1 bound to AP-1 (Fig. 5i). Moreover, NRIP1 and AP-1 chromatin immunoprecipitation sequencing experiments showed that AP-1 and NRIP1 binding sites were enriched near the transcription start sites (− 5 kb to 5 kb) of target genes (Additional file 2: Figure S6f). Interestingly, significant enrichment peaks for AP-1 and NRIP1 were detected at a genomic locus (chr 3: 4757428–4,757,590) upstream of the transcription start site of *EGOT* (Fig. 5j). Next, the binding of AP-1 and NRIP1 to the promoter region of *EGOT* was validated by ChIP-quantitative PCR (ChIP-PCR) (Fig. 5k). Taken together, our results indicated that NRIP1 physically interacts with AP-1 and transcriptionally represses the expression of *EGOT* in presence of E2 (Fig. 5l).

### *EGOT/ITPR1* expression is associated with a favorable prognosis and enhances paclitaxel sensitivity in human cancer

**Fig. 6:**
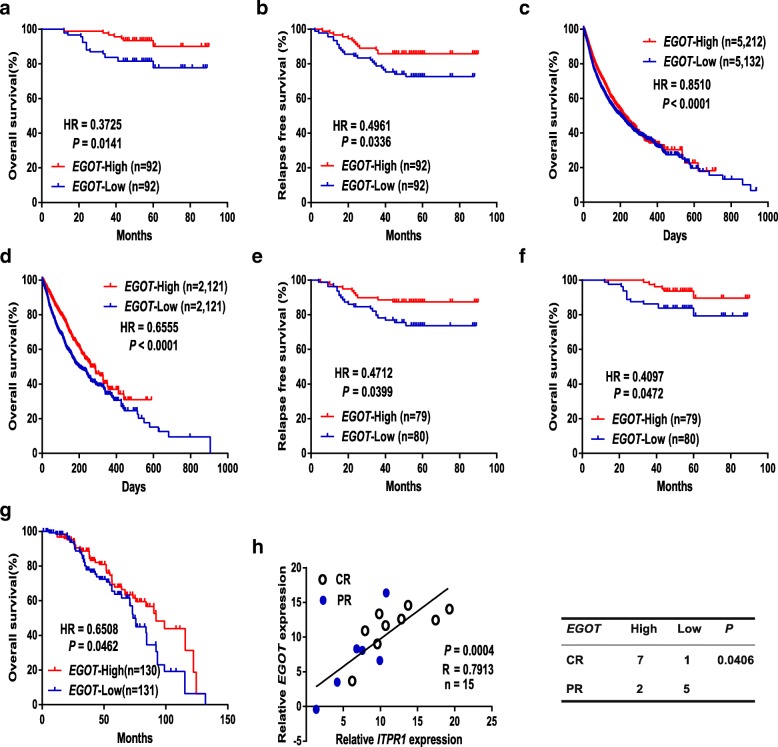
*EGOT*/*ITPR1* expression is associated with a favorable prognosis and enhances paclitaxel sensitivity in human cancer. **a**, **b** Kaplan-Meier analyses of the relationships between *EGOT* expression and OS (**a**) or RFS (**b**) in breast cancer patients in the HMUCC cohort. **c**, **d** Kaplan-Meier analyses of the relationships between *EGOT* expression and OS among 33 cancer types in TCGA (**c**) and ICGC cohorts (**d**). **e**, **f** Kaplan-Meier analyses of the relationships between *EGOT* expression and OS (**e**) or RFS (**f**) in breast cancer patients treated with paclitaxel in the HMUCC cohort. **g** Kaplan-Meier analyses of the relationship between *EGOT* expression and OS in breast cancer patients treated with paclitaxel in the TCGA cohort. **h** The correlations between *EGOT* expression and *ITPR1* mRNA expression were measured in 15 breast cancer patients treated with paclitaxel-containing adjuvant chemotherapy regimens (left). *EGOT* and *ITPR1* levels (normalized to *GAPDH*) were subjected to Pearson’s correlation analysis. Correlations between *EGOT* expression and a pathological response were determined by the chi-square test (right). A complete pathological response is denoted by CR, while a partial response is denoted by PR. The median expression level was used as the cut-off value. Patients with *EGOT* expression values below the 50th percentile were classified as *EGOT*-Low. Patients with *EGOT* expression values above the 50th percentile were classified as *EGOT*-High

The prognostic role of *EGOT* and *ITPR1* was finally examined in the HMUCC and public cohort data. High expression of *EGOT* was associated with favorable OS and RFS) in breast cancer patients, while loss of *ITPR1* indicated worse RFS in the HMUCC cohort (Fig. 6a and b; Additional file 2: Figure S7a). These results were then validated in 14,586 cancer patients in TCGA and ICGC human cancer cohorts, and patients with high expression levels of *EGOT* showed favorable OS and RFS in both of 33 human cancer types and breast cancer cohorts (Fig. 6c and d; Additional file 2: Figure S7b and c), while patients with high *ITPR1* expression showed favorable OS in the human cancer cohort (Additional file 2: Figure S7d and e).

Regarding paclitaxel sensitivity, breast cancer patients who were treated with paclitaxel and exhibited high expression levels of *EGOT* demonstrated better OS and RFS than patients with low expression levels of *EGOT* in the HMUCC cohort (Fig. 6e and f). Moreover, the results were further validated in TCGA breast cancer patients treated with paclitaxel, which showed that high expression of *EGOT* was also associated with favorable OS, in accordance with our study (Fig. 6g). Similarly, loss of *ITPR1* was associated with poor RFS in breast cancer patients treated with paclitaxel in the HMUCC cohort (Additional file 2: Figure S7f). More importantly, breast cancer patients with high expression of *EGOT* achieved a higher complete pathological response ratio than patients with low expression of *EGOT* when treated with paclitaxel-containing adjuvant chemotherapy regimens in the HMUCC cohort (Fig. 6h). Therefore, *EGOT*/*ITPR1* expression is associated with a favorable prognosis and enhances paclitaxel sensitivity in human cancer.

## Discussion

Regarding the function of Ai-lncRNA *EGOT*, we first reported that low levels of *EGOT* expression were significantly correlated with increased tumor size, lymph node metastasis, and Ki-67 expression in human breast cancer [14]. In this study, we identified an uncharacterized lncRNA, *EGOT*, and its roles in enhancing paclitaxel sensitivity by triggering autophagosome accumulation. Mechanistically, *EGOT* regulates ITPR1 expression *in cis* and *in trans*, leading to the activation of autophagy signaling. We also found that *EGOT* is transcriptionally regulated by various stress conditions that are associated with paclitaxel resistance. Overall, we provide compelling evidence that in response to stress, *EGOT* activates autophagy via ITPR1, which sensitizes paclitaxel cytotoxicity in human cancer (Additional file 2: Figure S8).

Several studies have investigated the role of macrophages in decreasing the cytotoxic effects of chemotherapy and demonstrated a therapeutic window for autophagy inhibition in cancer therapy and prevention [36, 37]. Paradoxically, a previous study showed that autophagy enhances paclitaxel-induced cell death and that reduced autophagy may contribute to clinical chemotherapeutic resistance in primary breast tumors [6]. Of note, paclitaxel blocks the activation of PI3K and Vps34 and inhibits the movement and maturation of autophagosomes to induce cell death [6]. In vitro, autophagosome accumulation sensitizes cells to paclitaxel toxicity [6]. Using in vivo and in vitro experiments in this study, we found that *EGOT* overexpression activated autophagy signaling, which sensitizes cells to paclitaxel, whereas *EGOT* knockdown has the opposite effect, in accordance with this previous study. Mechanistically, *EGOT* upregulates the expression of ITPR1, which promotes autophagosome accumulation and sensitizes cells to paclitaxel toxicity, thereby highlighting a novel function for these molecules.

Loss or low expression of autophagy-related genes, indicating a decrease of autophagic flux, was associated with poor prognosis [38, 39]. High expression of *EGOT* is significantly associated with favorable OS and RFS in patients in the HMUCC cohort and TCGA and ICGC public datasets. Furthermore, recent study showed that autophagy-related genes/proteins could be possible predictive markers for paclitaxel efficacy in the clinic [6]. High expression of *EGOT* is significantly associated with enhanced paclitaxel sensitivity in patients in the HMUCC cohort and TCGA and ICGC public datasets, while the lack of *EGOT* expression indicates resistance to paclitaxel therapy. In addition, high expression of *EGOT* indicated an increased complete pathological response ratio following treatment with paclitaxel-containing adjuvant chemotherapy regimens, in contrast to patients with low *EGOT* expression in the HMUCC cohort. However, paclitaxel-containing adjuvant chemotherapy sensitivity has only been evaluated in a small study cohort; thus, a multicenter clinical trial with a larger patient number will be performed in a future study to validate these results. Hence, our study provides a proof of concept showing that *EGOT* may be a predictive marker of the clinical efficacy of paclitaxel treatment.

LncRNAs can function *in cis* to regulate the expression of neighboring genes or *in trans* to carry out many roles by various modes [9]. Compared to other category of long non-coding RNAs, the function of Ai-lncRNAs including *EGOT* is still largely unknown. In this study, we clearly demonstrated that *EGOT* regulates ITPR1 expression both *in cis* and *in trans*. On one hand, *EGOT* regulates ITPR1 levels via a unique regulatory mechanism involving the formation of a *pre-ITPR1*/*EGOT* dsRNA that induces *pre-ITPR1* accumulation to increase ITPR1 protein expression *in cis*. However, we should mention that although it was initially proposed that lncRNA mainly functions to regulate neighboring gene transcription, other studies have shown that many lncRNAs do not exert such function [40, 41]. Thus, whether *EGOT* regulates any other neighboring gene transcription *in cis* awaits further investigation. Furthermore, *EGOT* recruits hnRNPH1 to promote ITPR1 expression *in trans*. hnRNPH1, a splicing factor, has been shown to regulate alternative splicing and polyadenylation [42, 43]. *EGOT* fragment 2 (324–645 nucleotides) in exon 1 was found to bind *pre-ITPR1* mRNA and the hnRNPH1 protein, thereby mediating alternative splicing of *pre-ITPR1* mRNA. hnRNPH1 has been shown to bind G-rich sequences interspersed with adenosines around exons [28, 42]. *ITPR1* contains 59 exons and multiple GGGA/C/G motifs distributed in these exons. Thus, we propose that hnRNPH1 mediates *pre-ITPR1* splicing in human cancer by binding these GGGA/C/G motifs. Taken together, these findings broaden the understanding of the autophagy-associated lncRNA landscape and provide novel insights into the exploration of the mechanism of autophagy regulation in human cancer.

Our data also show that *EGOT* is positively and negatively transcriptionally regulated by hypoxia and estrogen-related stress, respectively, in human cancer. Studies have shown that hypoxia can promote tumor invasion, metastasis, autophagy, and angiogenesis, can regulate tumor metabolism and is closely related to the poor prognosis of cancers [44, 45]. Hypoxia is associated with the expression of numerous noncoding RNAs, including miRNAs and lncRNAs [46]. In this study, *EGOT* was induced in vitro by subjecting different cancer cell lines to hypoxia. Apart from its positive regulation by hypoxia, *EGOT* can be transcriptionally repressed by estrogen. Estrogen, a paracrine mediator throughout life, is an important factor in tumorigenesis in humans. Estrogen binds to estrogen receptors to control vast gene networks that are involved in glycolysis, glutaminolysis, oxidative phosphorylation, nutrient sensing and biosynthesis pathways in cancer [47]. Several lncRNAs have been shown to be regulated by estrogen in breast cancer [22, 48]. In this study, *EGOT* was found to be a direct target of AP-1/NRIP1-mediated transcriptional repression complex in the presence of estrogen. Previous studies have shown that NRIP1 is an obligate co-factor of the estrogen receptor, and germline single-nucleotide polymorphisms (SNPs) near NRIP1 have been associated with ER-positive breast cancer [49]. Estrogen mediates NRIP1 induction, which subsequently interacts with estrogen receptor AP-1 complexes to directly repress adjacent target genes, including *BCAS4*, *IRX4*, *GUSB* and *MUC1* [35]. In this study, *EGOT* was inhibited by estrogen at physiological concentrations (0–10^− 9^ M), which led to a gradual decrease in *EGOT* expression; meanwhile NRIP1 expression was gradually increased with time. Knockdown of NRIP1 reversed the estrogen-mediated transcriptional repression of *EGOT* expression. Interestingly, a previous study reported that the expression of ITPR1 protein decreased in an estrogen receptor-dependent manner and that the growth of MCF7 cells induced by estrogen was sensitive to pharmacological inhibitors of ITPR1 [16]. Our study provides a proof of concept indicating that *EGOT* is transcriptionally repressed by the AP-1/NRIP1 complex in the presence of estrogen and that this axis may send regulatory feedback signals to repress the excessive activation of autophagy.

In conclusion, these findings broaden comprehensive understanding of the biology function of Ai-lncRNAs. Moreover, our findings demonstrate that *EGOT* my act as a clinical biomarker of paclitaxel response and that proper regulation of *EGOT* may be a novel synergistic strategy for enhancing paclitaxel sensitivity, thereby enhancing its clinical benefits for cancer patients.

## Additional files

Additional file 1: **Table S1.** Lists of primers. Lists including PCR primers used in this study, primers used for shRNAs and siRNA sequences. **Table S2.** Details of the gene sequence probe sets. **Table S3.** Guilt-by-association analysis in breast cancer data from TCGA. **Table S4.** Pan-cancer data of 33 cancer contexts in TCGA. All cancer IDs and patient numbers are listed. **Table S5.** Protein mass spectrometry analysis in MDA-MB-231. RNA pull-down assays using the Flag-MS2bp-MS2bs-based system (Additional file 2: Figure S4F), followed by mass spectrometry in MDA-MB-231 cells. Raw data listing all identified proteins and all peptides from each sample. (ZIP 9889 kb)

Additional file 2: **Figure S1.** *EGOT* enhances paclitaxel sensitivity in human cancer. **Figure S2.** *EGOT* is positively related to ITPR1 and induces ITPR1 expression. **Figure S3.** *EGOT* induces autophagy to enhance paclitaxel sensitivity through ITPR1. **Figure S4.** *EGOT* regulates ITPR1 expression *in cis* and *in trans* in human cancer. **Figure S5.** *EGOT* is transcriptionally regulated by stress stimuli in human cancer. **Figure S6.** *EGOT* is directly repressed by the NRIP1/AP-1 complex. **Figure S7.** *EGOT*/*ITPR1* is associated with a favorable prognosis and enhances paclitaxel sensitivity in human cancer. **Figure S8.** Schematic illustration of the possible mechanisms involved in this study. (ZIP 9814 kb)

## Abbreviations

Ai-lncRNA: Antisense intronic long noncoding RNA
AP-1: Activator protein 1
CCLE: Cancer Cell Line Encyclopedia
ChIP: Chromatin Immunoprecipitation
Co-IP: Co-Immunoprecipitation
CQ: Chloroquine
DFS: Disease free survival
EBSS: Earle's Balanced Salt Solution
*EGOT*: Eosinophil granule ontogeny
ER: endoplasmic reticulum
ERE: Estrogen response element
hnRNPH1: Heterogeneous nuclear ribonucleoprotein H1
ICGC: International Cancer Genome Consortium
ITPR1: Inositol 1,4,5-trisphosphate receptor 1
LC3B: Microtubule associated protein 1 light chain 3 beta
LncRNA: Long noncoding RNA
NRIP1: Nuclear receptor interacting protein 1
RIP: RNA-binding protein immunoprecipitation
TCGA: The Cancer Genome Atlas
