# Supplementary material for: Ai-lncRNA EGOT enhancing autophagy sensitizes paclitaxel cytotoxicity via upregulation of ITPR1 expression by RNA-RNA and RNA-protein interactions in human cancer
Source: Mol Cancer. 2019 Apr 18;18:89. doi: 10.1186/s12943-019-1017-z (PMC6471868; doi:10.1186/s12943-019-1017-z)
Supplement: Supplementary file 1 — Table S1. Lists of primers. Lists including PCR primers used in this study, primers used for shRNAs and siRNA sequences. Table S2. Details of the gene sequence probe sets. Table S3. Guilt-by-association analysis in breast cancer data from TCGA. Table S4. Pan-cancer data of 33 cancer contexts in TCGA. All cancer IDs and patient numbers are listed. Table S5. Protein mass spectrometry analysis in MDA-MB-231. RNA pull-down assays using the Flag-MS2bp-MS2bs-based system (Additional file 2: Figure S4F), followed by mass spectrometry in MDA-MB-231 cells. Raw data listing all identified proteins and all peptides from each sample. (ZIP 9889 kb) [file 12943_2019_1017_MOESM1_ESM.zip › Table S2.docx]

**Place probesets on gene sequence**

Label extension probe is red and block liquid probe is blue.

>gi|168480144|ref|NM_001101| Homo sapiens actin beta (ACTB), mRNA. [Homo sapiens] PRI 06-OCT-2016

accgccgagaccgcgtccgccccgcgagcacagagcctcgcctttgccgatccgccgcccgtccacacccgccgccagct
caccatggatgatgatatcgccgcgctcgtcgtcgacaacggctccggcatgtgcaaggccggcttcgcgggcgacgatg
ccccccgggccgtcttcccctccatcgtggggcgccccaggcaccagggcgtgatggtgggcatgggtcagaaggattcc
tatgtgggcgacgaggcccagagcaagagaggcatcctcaccctgaagtaccccatcgagcacggcatcgtcaccaactg
ggacgacatggagaaaatctggcaccacaccttctacaatgagctgcgtgtggctcccgaggagcaccccgtgctgctga
ccgaggcccccctgaaccccaaggccaaccgcgagaagatgacccagatcatgtttgagaccttcaacaccccagccatg
tacgttgctatccaggctgtgctatccctgtacgcctctggccgtaccactggcatcgtgatggactccggtgacggggt
cacccacactgtgcccatctacgaggggtatgccctcccccatgccatcctgcgtctggacctggctggccgggacctga
ctgactacctcatgaagatcctcaccgagcgcggctacagcttcaccaccacggccgagcgggaaatcgtgcgtgacatt
aaggagaagctgtgctacgtcgccctggacttcgagcaagagatggccacggctgcttccagctcctccctggagaagag
ctacgagctgcctgacggccaggtcatcaccattggcaatgagcggttccgctgccctgaggcactcttccagccttcct
tcctgggcatggagtcctgtggcatccacgaaactaccttcaactccatcatgaagtgtgacgtggacatccgcaaagac
ctgtacgccaacacagtgctgtctggcggcaccaccatgtaccctggcattgccgacaggatgcagaaggagatcactgc
cctggcacccagcacaatgaagatcaagatcattgctcctcctgagcgcaagtactccgtgtggatcggcggctccatcc
tggcctcgctgtccaccttccagcagatgtggatcagcaagcaggagtatgacgagtccggcccctccatcgtccaccgc
aaatgcttctaggcggactatgacttagttgcgttacaccctttcttgacaaaacctaacttgcgcagaaaacaagatga
gattggcatggctttatttgttttttttgttttgttttggttttttttttttttttggcttgactcaggatttaaaaact
ggaacggtgaaggtgacagcagtcggttggagcgagcatcccccaaagttcacaatgtggccgaggactttgattgcaca
ttgttgtttttttaatagtcattccaaatatgagatgcgttgttacaggaagtcccttgccatcctaaaagccaccccac
ttctctctaaggagaatggcccagtcctctcccaagtccacacaggggaggtgatagcattgctttcgtgtaaattatgt
aatgcaaaatttttttaatcttcgccttaatacttttttattttgttttattttgaatgatgagccttcgtgccccccct
tcccccttttttgtcccccaacttgagatgtatgaaggcttttggtctccctgggagtgggtggaggcagccagggctta
cctgtacactgacttgagaccagttgaataaaagtgcacaccttaaaaatgaaaaaaaaaaaaaaaaaaaaaaaaaaaaa
aaaaaaaaaaaa

>gi|161333876|ref|NR_004428| Homo sapiens eosinophil granule ontogeny transcript (non-proteincoding) (EGOT), long non-coding RNA. [Homo sapiens] PRI 24-DEC-2015

caacttctgggcaaggcagaggtgggtttggctttttaaaaattttttcagcctgtcctcatggaactacatattctttt
ctaagaacttttcatcctaacctccctactcacatcttctaagtgtctctgctctggtgggaatgtgatggacaacacag
agccatctcagaagcctctgtggccaccaccaggccggccagggtgcagggggccactccctgggcagccatagggttct
cagcaaggtgcattcgtcgtccctgctgagaatctgatggggcagcatttttttttttaattaaatgcaagctgagtcat
ttcaacctgcaaccttcaggtaacaggagttacccaagctccaggaattatgattgtggggtaaacccattctcttgttt
tcttgcggttctattttataacgcactagaggagacagagacgtcattgcttcacccagggcaagaggacaaaggaggat
gcatggaaatacaaacagcccttctcctccaggccataccgactgtccaactagcaacagacttcacctggttttggagc
aatctgaatttggaatatgccagagaaaacttctatcaggcaagatggaagactcctaggataggtctctctcatggagg
gattggcacgatggtaagagtccagtggatggaaaaggcctcctccttaaagatggtgatggaaacgagcactgcacagg
gaaacacaaatcaggtcccctgaaatccagcccaacctggccagaccctccagtgcccatcagggcttatgaacagggtg
cttcagtgtctttgttaggggtggttaaaaaggagcacgtgcttataggggatgctgctgagctccatgattttgacttc
ctgtctaacctgttgatgctacaaaactcttttaaaaacagttaccatgggaacttttccctagcaagcattcttcaaga
aacgttacaggtgctggtgaattcaatgcccttggtgtcaataagcttttgctgagtcttagaatgtgtggcttgaactg
agaggactttcaaacagataatgcgaagtttccaatgcaaatgtcttcggggttaagtgtcagtgaagtgcagtggaagg
tagggggataaaatgatctcagtgtatatggaggccccacttaagcattcaaaaaattctatatacagtgttttcaatgt
attatgtatcagcaaaacaaaataccccatagggctataatcagctcagggggtcacctcagcaagagatgctctggcta
tcactcctactacgggatttcctctctcatcagaaccagtaggtggtcaaaaccacaataatgcataaatgaaggcaggg
aattgggtatccaatgtgagcaactgaaaaggtgccatgtattagctatttgccattatggttctgcaagtgctcagatg
actcataaaatgaccagaacaaaaaaaaaaaaaaaaaaaaaaaaaaaaaaaaaaaaaaaaaaaaaaaaaaaaaaaaaaaa
aaaaaaaaa

>gi|269954691|ref|NM_002222| Homo sapiens inositol 1,4,5-trisphosphate receptor type 1 (ITPR1), mRNA. [Homo sapiens] PRI 26-AUG-2016

gcagtaaccatgtggatgtgctgctgaagcgtttcctcaagctcgctggggtgggaggagaggaggaggaggaggtggtg
gtggaggaggaggcagggggtggagagagagaaagcgcacgccgagaggaggtgtgggtgttccgcttccatcctaacgg
aacgagctccctcttcgcggacatgggattacccagcggctgctaacccctctcctcgccctgctcccccaaaccggcgt
ggctccccgggcaccaaggagctgactacagaggagcaggatttgcacccctcgctgggcttgctttggcaacagagtgc
ctgacccaggtcaggattttcaagaaagacatgtctgacaaaatgtctagcttcctacatattggagacatttgttctct
gtacgcggagggatcgacaaatggatttattagcaccttgggcctggttgatgatcgttgtgttgtacagccagaaaccg
gggaccttaacaatccacctaagaaattcagagactgcctctttaagctatgtcccatgaaccgctactctgcccaaaag
cagttctggaaagccgctaagcctggggccaacagcaccacagacgcagtgctactcaacaaactgcaccacgctgcaga
cttggaaaagaagcagaatgagacagaaaacaggaaattgctggggaccgtaatccagtatggcaatgtgatccagctcc
tgcatttgaaaagtaataaatacctaacagtgaataagaggcttcctgctctgttggagaagaatgccatgagagtcaca
ttggacgaggctggaaatgaagggtcctggttttatattcagccattctacaagctgcgatccattggagacagcgtggt
cataggtgacaaggtggttctgaaccccgtcaatgctggtcagcccctacatgctagcagccatcaactggtagataacc
caggctgcaatgaggtcaattccgtcaactgcaatacaagctggaaaatagtccttttcatgaaatggagtgataacaaa
gacgacatattaaaggggggtgacgtggtgaggctgtttcatgctgagcaggagaagtttctcacctgtgacgaacacag
gaagaagcagcacgtcttcctgagaaccacgggccggcagtcggccacatctgccaccagttcaaaagccctgtgggagg
tggaggtggtccagcatgacccatgtcggggcggagcagggtattggaacagccttttccgtttcaagcatctggccacg
gggcattacttggcagcagaggtggaccctgatcaggacgcctctcgaagtaggttgcggaatgcccaagaaaagatggt
atactccctggtctctgtgcctgaaggcaatgacatctcctccattttcgagctagatcccaccactctgcgtggaggtg
acagccttgtcccaaggaactcttatgttcggctcagacacctatgtactaatacctgggttcacagcacaaatattcct
attgacaaggaagaagaaaagcccgtgatgctgaaaattggcacctctcctgtgaaggaggataaggaagcatttgccat
agttccggtttctcctgctgaagttcgggacctggactttgccaatgatgccagcaaggtgctgggctccattgctggga
agctagagaagggcaccatcacccagaatgaaaggaggtctgtaaccaagctgctagaagatttggtttacttcgtcact
ggtggaactaattctggtcaagatgttctcgaagttgtcttctccaagcccaacagagaacggcagaaactgatgagaga
acagaatattctcaagcagatcttcaagttgttacaagccccattcacagactgcggtgatggcccaatgcttcggctgg
aagagctcggggaccagcggcacgctcctttcagacacatctgccggctctgctacagggtgctgagacactcgcagcaa
gactacaggaagaaccaggagtatatagccaagcagtttggcttcatgcagaagcagattggctatgatgtgttggctga
agacactatcactgccctgctccacaataatcggaaactcctggaaaaacacattaccgcggcagagattgacacatttg
tcagcctggtgcgaaagaacagggagcccagattcttagattacctctccgacctctgtgtctccatgaacaaatcaatt
ccagtgacccaggaactgatatgtaaagctgtgctgaaccccaccaacgctgacatcctgattgagaccaagttggttct
ttctcgttttgaatttgaaggtgtctcttccactggagagaatgctctggaggcaggagaagacgaggaagaggtgtggc
tgttttggagggacagcaacaaagagattcgcagcaagagtgtgagggaattggctcaggatgctaaagaagggcagaag
gaggaccgagacgttctcagctactacagatatcagctgaacctctttgcgaggatgtgtctggaccgccaatacctggc
catcaacgaaatctcaggccagctggatgtcgatctcattctccgctgcatgtctgacgagaacctgccctatgacctca
gggcgtccttctgccgcctcatgcttcacatgcatgtggaccgagatccccaggaacaagtcacccccgtgaaatatgcc
cgcctctggtcggagattccctcggagatcgccattgacgactatgatagtagtggagcttccaaagatgaaattaagga
gagatttgctcagaccatggagtttgtggaggagtatttaagagatgtggtttgtcagaggttccctttctctgataaag
agaagaataagcttacgtttgaggttgtaaatttagctaggaatctcatatactttggtttctacaacttctctgacctt
ctacgattaactaagatccttctggccatattggactgtgtacatgtgacaacaatcttccccattagcaagatggcgaa
aggagaagagaataaaggcagtaacgtgatgagatctattcatggcgtgggagagctgatgacccaggtggtgctccggg
gaggaggctttttgcccatgactcccatggctgctgcccctgaaggcaatgtgaagcaggcagagcctgagaaggaggac
atcatggtcatggacaccaagctgaagatcattgagatactccagtttattttgaatgtgaggttggattataggatctc
ctgcctcctgtgtatatttaagcgagagtttgatgaaagcaattcccagacttcagaaacatcctccggaaacagcagcc
aagaagggccaagtaatgtaccaggtgctcttgactttgaacacattgaagaacaagcagaaggcatctttggaggaagt
gaggagaacaccccactggacttggatgaccacggcggcagaacctttctccgtgtcctgctccacttgacgatgcatga
ctacccacccctggtgtcaggggccctgcagctcctcttccggcacttcagccagaggcaggaggtgctccaggccttca
aacaggttcaactgctggttaccagccaagatgtggacaactacaaacagatcaaacaagacttggatcaactgaggtcc
atcgtggaaaagtcagagctttgggtgtacaaagggcagggccccgatgagactatggatggtgcatctggagaaaatga
acataagaaaacggaggagggaaataacaagccacaaaagcatgaaagcaccagcagctacaactacagagtggtcaaag
agattttgattcggcttagcaaactctgtgttcaagagagtgcctcagtgagaaagagcaggaagcagcaacagcgtctg
ctccggaacatgggcgcgcacgccgtggtgctggagctgctgcagattccctatgagaaggccgaagataccaagatgca
agagataatgaggttggctcatgaatttttgcagaatttctgcgcaggcaaccagcagaatcaagctttgctacataaac
acataaacctgtttctcaacccagggatcctggaggcagtaaccatgcagcacatcttcatgaacaatttccagctttgc
agtgagatcaacgagagagttgttcagcacttcgttcactgcatagagactcacggtcggaatgtccagtatataaagtt
cttacagacaattgtcaaggcagaagggaaatttattaaaaaatgccaagacatggttatggccgagctggtcaattcgg
gagaggatgtcctcgtgttctacaacgacagagcctctttccagactctgatccagatgatgcggtcagaacgggatcgg
atggatgagaacagccctctcatgtaccacatccacttggtcgagctcctggctgtgtgcacggagggtaagaatgtcta
cacagagatcaagtgcaactccctgctcccgctggatgacatcgttcgcgtggtgacccacgaggactgcatccctgagg
ttaaaattgcatacattaacttcctgaatcactgctatgtggatacagaggtggaaatgaaggagatttataccagcaat
cacatgtggaaattgtttgagaatttccttgtagacatctgcagggcctgtaacaacactagtgacaggaaacatgcaga
ctcgattttggagaagtatgtcaccgaaatcgtcatgagtattgttactactttcttcagctctcccttctcagaccaga
gtacgactttgcagactcgccagcctgtctttgtgcaactgctgcaaggcgtgttcagggtttaccactgcaactggtta
atgccaagccaaaaagcctccgtggagagctgtattcgggtgctgtctgatgtagccaagagccgggccattgccattcc
cgtggacctggacagccaagtcaacaacctctttctcaagtcccacagcattgtgcagaaaacagccatgaactggcggc
tctcagcccgcaatgccgcacgcagggactctgttctggcagcttccagagactaccggaatatcattgagagattgcag
gacatcgtctccgcgctggaggaccgtctcaggcccctggtgcaggcagagttatctgtgctcgtggatgttctccacag
acccgagctgcttttcccagagaacacagacgccagaaggaaatgtgaaagtggcggtttcatttgcaagttaataaagc
atacaaaacagctgctagaagaaaatgaagagaagctctgcattaaggtcctacagaccctgagggaaatgatgaccaaa
gatagaggctatggagaaaagggtgaggcgctcaggcaagttctggtcaaccgttactatggaaacgtcagaccttcggg
acgaagagagagccttaccagctttggcaatggcccactgtcagcaggaggacccggcaagcccgggggaggagggggag
gttccggatccagctctatgagcaggggtgagatgagtctggccgaggttcagtgtcaccttgacaaggagggggcttcc
aatctagttatcgacctcatcatgaacgcatccagtgaccgagtgttccatgaaagcattctcctggccattgcccttct
ggaaggaggcaacaccaccatccagcactcctttttctgtcgcttgacagaagataagaagtcagagaaattctttaagg
tgttttatgaccggatgaaggtggcccagcaagaaatcaaagcaacagtgacagtgaacaccagtgacttgggaaataaa
aagaaagacgatgaggtagacagggatgccccatcacggaaaaaagctaaagagcccacaacacagataacagaagaggt
ccgggatcagctcctggaggcctccgctgccaccaggaaagccttcaccactttcaggagggaggctgatcccgacgacc
actaccagcctggagagggcacccaggccactgccgacaaggccaaggacgacctggagatgagcgcggtcatcaccatc
atgcagcccatcctccgcttccttcagctcctgtgtgaaaaccacaaccgagacctgcagaacttcctccgttgccaaaa
taacaagaccaactacaatttggtatgtgagaccctgcagtttctggactgtatttgtggaagcacaactggaggccttg
gtcttctgggcttgtatataaatgaaaagaacgtagcgcttatcaaccaaaccctggaaagtctgaccgaatactgtcaa
ggaccttgccatgagaaccagaactgcatagccacccatgaatccaatggcattgacatcatcacagccctgatcctcaa
tgatatcaatcctttgggaaagaagaggatggaccttgtgttagaactgaagaacaatgcctcgaagttgctcctggcca
tcatggaaagcaggcacgacagtgaaaacgcagagaggatactttataacatgaggcccaaggaactggtggaagtgatc
aagaaagcctacatgcaaggtgaagtggaatttgaggatggagaaaacggtgaggatggggcggcgtcccccaggaacgt
ggggcacaacatctacatattagcccatcagttggctcggcataacaaagaacttcagagcatgctgaaacctggtggcc
aagtggacggagatgaagccctggagttttatgccaagcacacggcgcagatagagattgtcagattagaccgaacaatg
gaacagatagtctttcccgtgcccagcatatgtgaattcctaaccaaggagtcaaaactacgaatttactatactacaga
gagagacgaacaaggcagcaaaatcaatgatttctttctgcggtctgaagacctcttcaatgaaatgaattggcagaaga
aactgagagcccagcccgtgttgtactggtgtgcccgcaacatgtctttctggagcagcatttcgtttaacctggccgtc
ctgatgaacctgctggtggcgtttttctacccgtttaagggagtccgaggaggaaccctggagccccactggtcgggact
cctgtggacagccatgctcatctctctggccatcgtcattgccctccccaagccccatggcatccgggccttaattgcct
ccacaattctacgactgatattttcagtcgggttacaacccacgttgtttcttctgggcgctttcaatgtatgcaataaa
atcatctttctaatgagctttgtgggcaactgtgggacattcacaagaggctaccgagccatggttctggatgttgagtt
cctctatcatttgttgtatctggtgatctgtgccatggggctctttgtccatgaattcttctacagtctgctgctttttg
atttagtgtacagagaagagactttgcttaatgtcattaaaagtgtcactcgcaatggacggtccatcatcctgacagca
gttctggctctgatcctcgtttacctgttctcaatagtgggctatcttttcttcaaggatgactttatcttggaagtaga
taggctgcccaatgaaacagctgttccagaaaccggcgagagtttggcaagcgagttcctgttctccgatgtgtgtaggg
tggagagtggggagaactgctcctctcctgcacccagagaagagctggtccctgcagaagagacggaacaggataaagag
cacacatgtgagacgctgctgatgtgcattgtcactgtgctgagtcacgggctgcggagcgggggtggagtaggagatgt
actcaggaagccgtccaaagaggaacccctgtttgctgctagagttatttatgacctcttgttcttcttcatggtcatca
tcattgttcttaacctgatttttggggttatcattgacacttttgctgacctgaggagtgagaagcagaagaaggaagag
atcttgaagaccacgtgctttatctgtggcttggaaagagacaagtttgacaacaagactgtcacctttgaagagcacat
caaggaagaacacaacatgtggcactatctgtgcttcatcgtcctggtgaaagtaaaggactccaccgaatatactgggc
ctgagagttacgtggcagaaatgatcaaggaaagaaaccttgactggttccccaggatgagagccatgtcattggtcagc
agtgattctgaaggagaacagaatgagctgagaaacctgcaggagaagctggagtccaccatgaaacttgtcacgaacct
ttctggccagctgtcggaattaaaggatcagatgacagaacaaaggaagcagaaacaaagaattggtcttctaggacatc
ctcctcacatgaatgtcaacccacaacaaccagcataagcaaatgaaagaaaggaattgtatttaccttttataattatt
attagtgtgggtatggctaatgagttctgattcacccacgaaggttacatttatgctgaatacatttgtaaatactcagt
tttatactgtatgtatatgattgctactctaaaggtttggatatatgtattgtaattagaattgttggcatgatgacatt
tcatttgtgccaaaaatattaaaaatgccttttttggaaggactaacagaaagcacctgatttgcacttgaaccagatta
tagatttaaaagtatatgacatgtattttgtatttaaaactagaatagccagtatttatgttttttataaaactgtgcaa
tacgaattatgcaatcacaatacatttgtagctcccgagtgtcctaaagggagtgcacttctttgaagctggtgtgttaa
tactatgtaataaatggttaactttcaaatgatgctgctgccaaaattatattaatagtgagtttcaggcccctgggcat
tttgtaccatgtaattatcctctggtgatgctgtttctcgttagtggcagtagtgcctccgtctcctagtgataatgctc
caagtctatgaactgttaaatcagcattcattttaagaaaagcaactttagtttcaaagatacttttaagcttctaaatt
gatcatttaaactatttctttaaataagagagccaaattagaggctcatactttagcttgtgaagaagataatgaatttt
ttaaagggaactttctatgcaatgttcaggataaatgcatactgctggccaatcagtgtcatctcctgggtaaattttga
tgtcgcattataaagacatgcataattgatggtttctagattatctagtccaaacaatagagtttattttttcttcatct
gaaccaacatgctacagtagctaagaagtattaaaactatatacatccatataaagatgaaatatgaactatctcattag
aagtcatagttgaccacagacatgttattcttctgaaagagccacattttggttttatttcttgtcacatgatttctttt
cttgatggatgaaaaatatgaaaggaaacttttatatctgttgcctagttttgtacatggatctcattttacaagagaat
ctctctgcaaaaaaaaaaaaaacagtttaaaaatgcattgaaagcagagttctgaaatgagtaaagtttgtaaatgcata
tataaaaatatttaataaatgatgcagaatatacagtgactggttggtggctttcatttggcatttgtgacttaactgct
attccatttatgtactttctttaggatcagtttgaagtacagtcggtttgattaccaggttaattcagaaaatgtttacc
tgattatttaaaaaaacacttgctgttgttatgacagctctgaggagaattattagaacaaacattggggagtatttagt
acaacattcattcattcttctggattagtgactgccagccaggctgtcgggagcgccctactaaatctcctgtctcttct
gcttttactgggggatctttaatttgctgtgtgccatgtggaagtatcgggaa
