# Supplementary figures and images for: Ai-lncRNA EGOT enhancing autophagy sensitizes paclitaxel cytotoxicity via upregulation of ITPR1 expression by RNA-RNA and RNA-protein interactions in human cancer
Source: Mol Cancer. 2019 Apr 18;18:89. doi: 10.1186/s12943-019-1017-z (PMC6471868; doi:10.1186/s12943-019-1017-z)

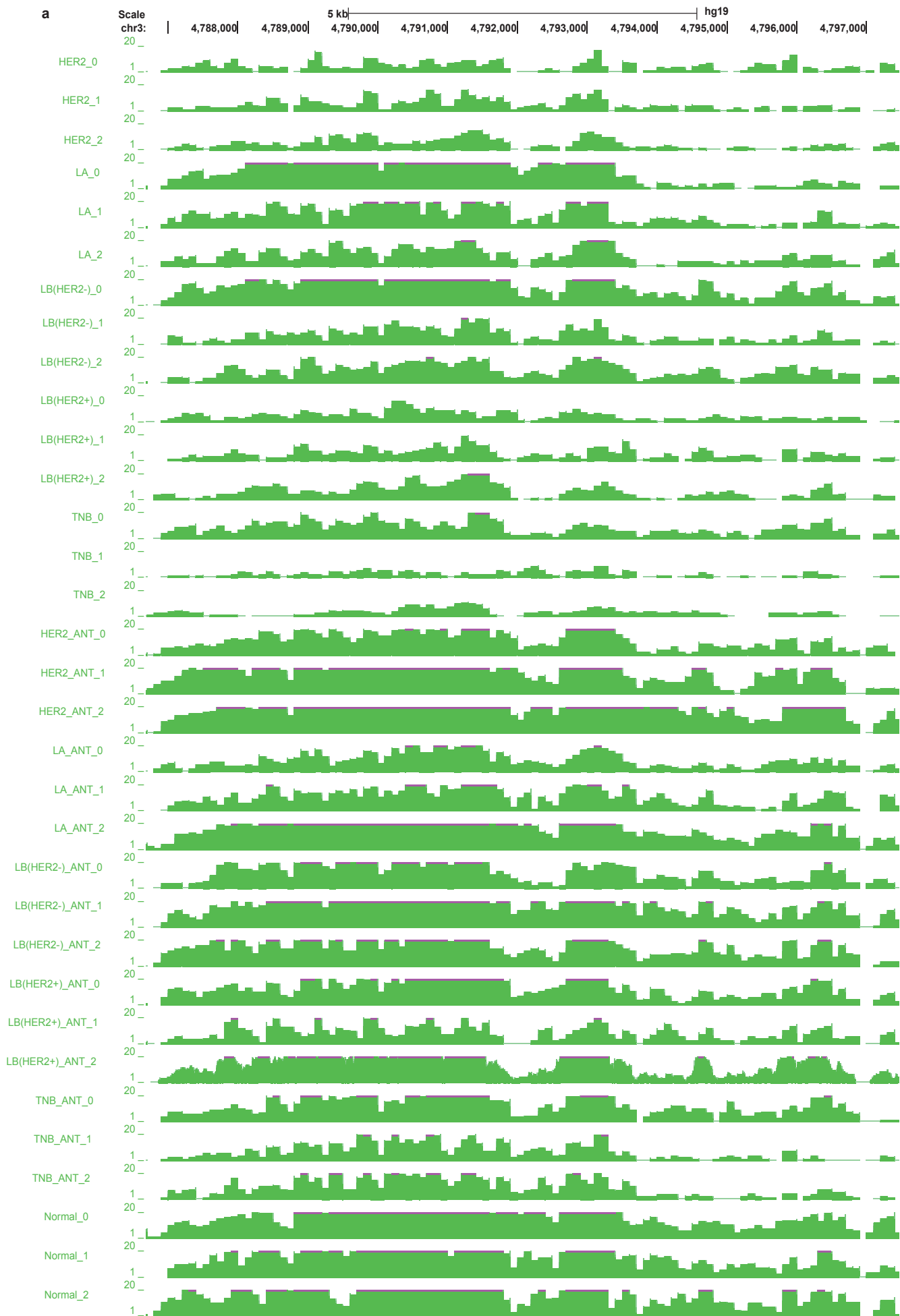

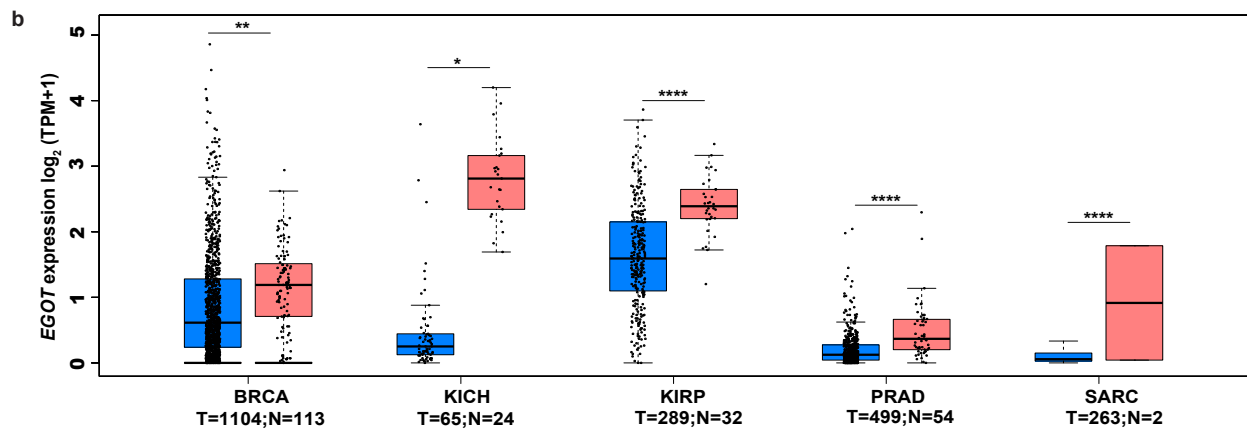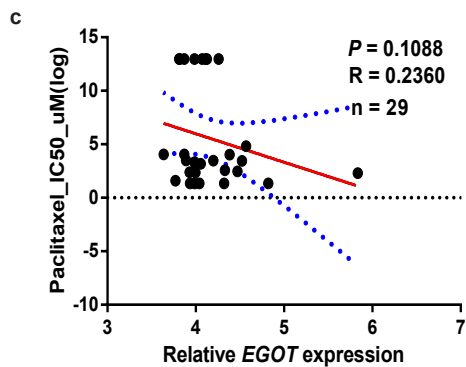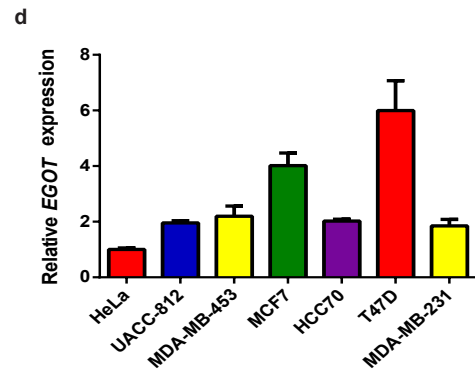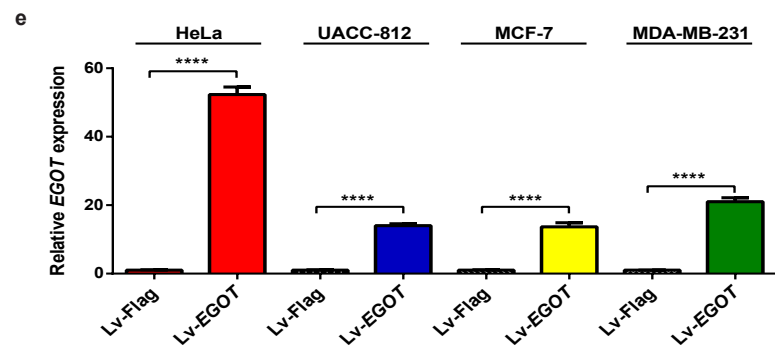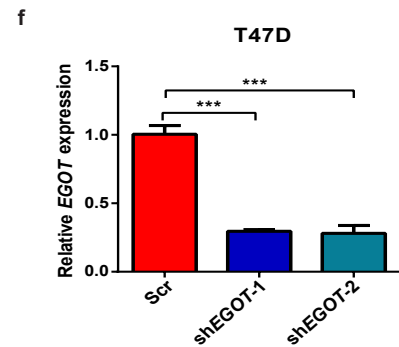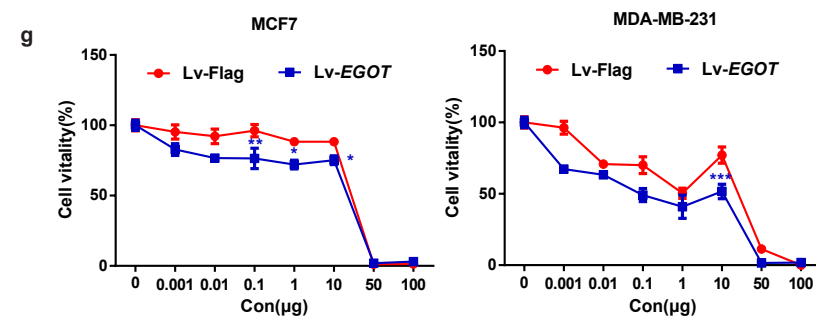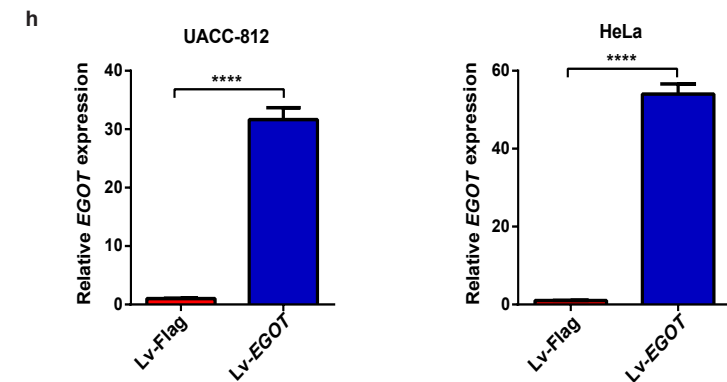

i

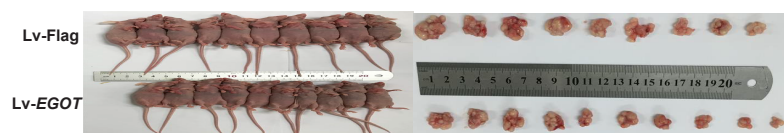

j

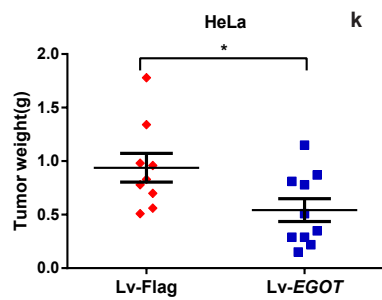

k

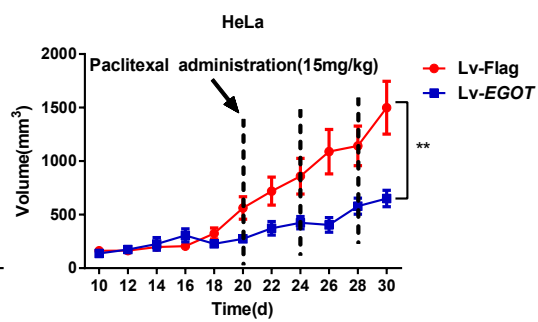

Supplement: Supplementary file 1 — Table S1. Lists of primers. Lists including PCR primers used in this study, primers used for shRNAs and siRNA sequences. Table S2. Details of the gene sequence probe sets. Table S3. Guilt-by-association analysis in breast cancer data from TCGA. Table S4. Pan-cancer data of 33 cancer contexts in TCGA. All cancer IDs and patient numbers are listed. Table S5. Protein mass spectrometry analysis in MDA-MB-231. RNA pull-down assays using the Flag-MS2bp-MS2bs-based system (Additional file 2: Figure S4F), followed by mass spectrometry in MDA-MB-231 cells. Raw data listing all identified proteins and all peptides from each sample. (ZIP 9889 kb) [file 12943_2019_1017_MOESM1_ESM.zip › Figure S1.pdf]

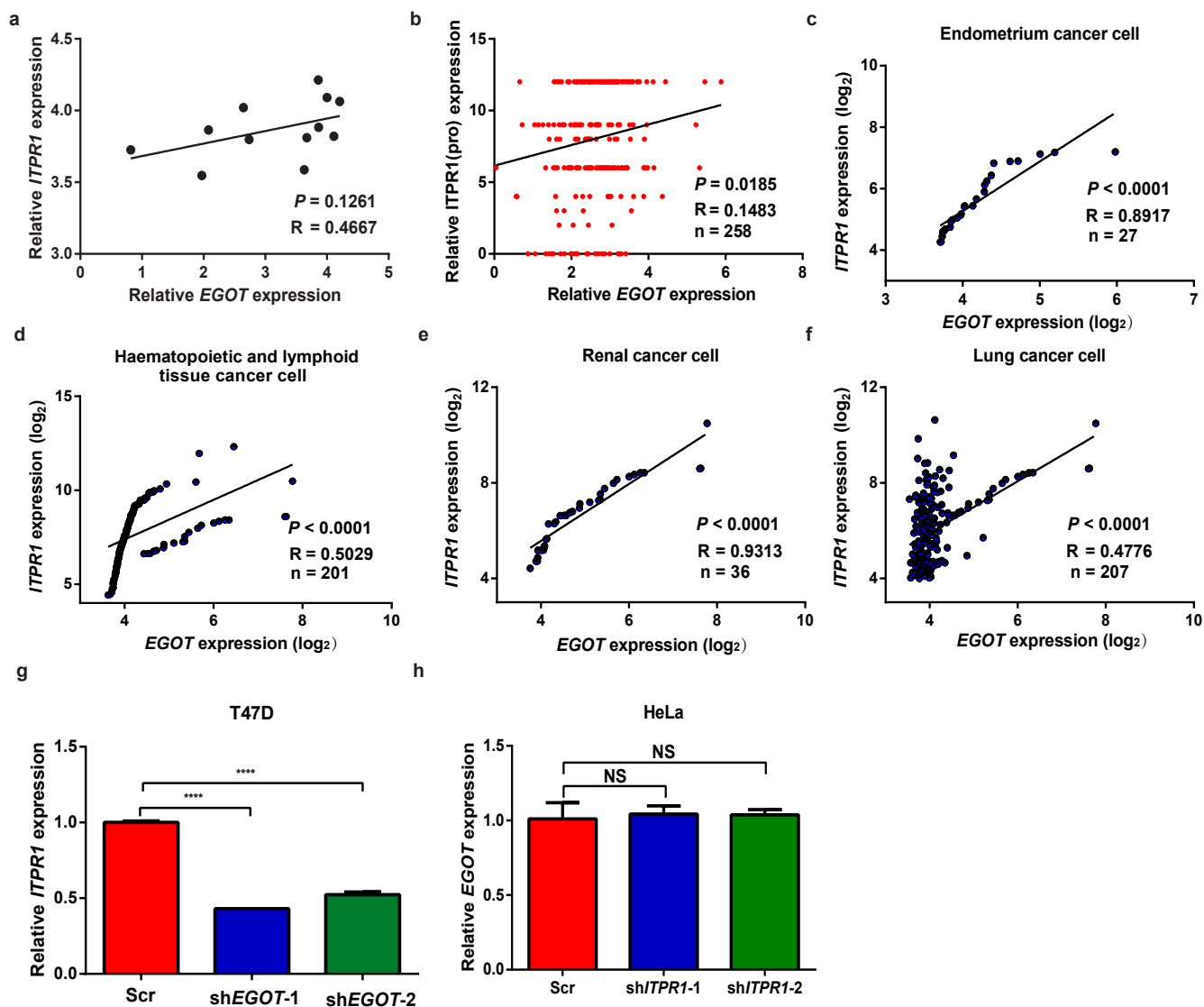

Supplement: Supplementary file 1 — Table S1. Lists of primers. Lists including PCR primers used in this study, primers used for shRNAs and siRNA sequences. Table S2. Details of the gene sequence probe sets. Table S3. Guilt-by-association analysis in breast cancer data from TCGA. Table S4. Pan-cancer data of 33 cancer contexts in TCGA. All cancer IDs and patient numbers are listed. Table S5. Protein mass spectrometry analysis in MDA-MB-231. RNA pull-down assays using the Flag-MS2bp-MS2bs-based system (Additional file 2: Figure S4F), followed by mass spectrometry in MDA-MB-231 cells. Raw data listing all identified proteins and all peptides from each sample. (ZIP 9889 kb) [file 12943_2019_1017_MOESM1_ESM.zip › Figure S2.pdf]

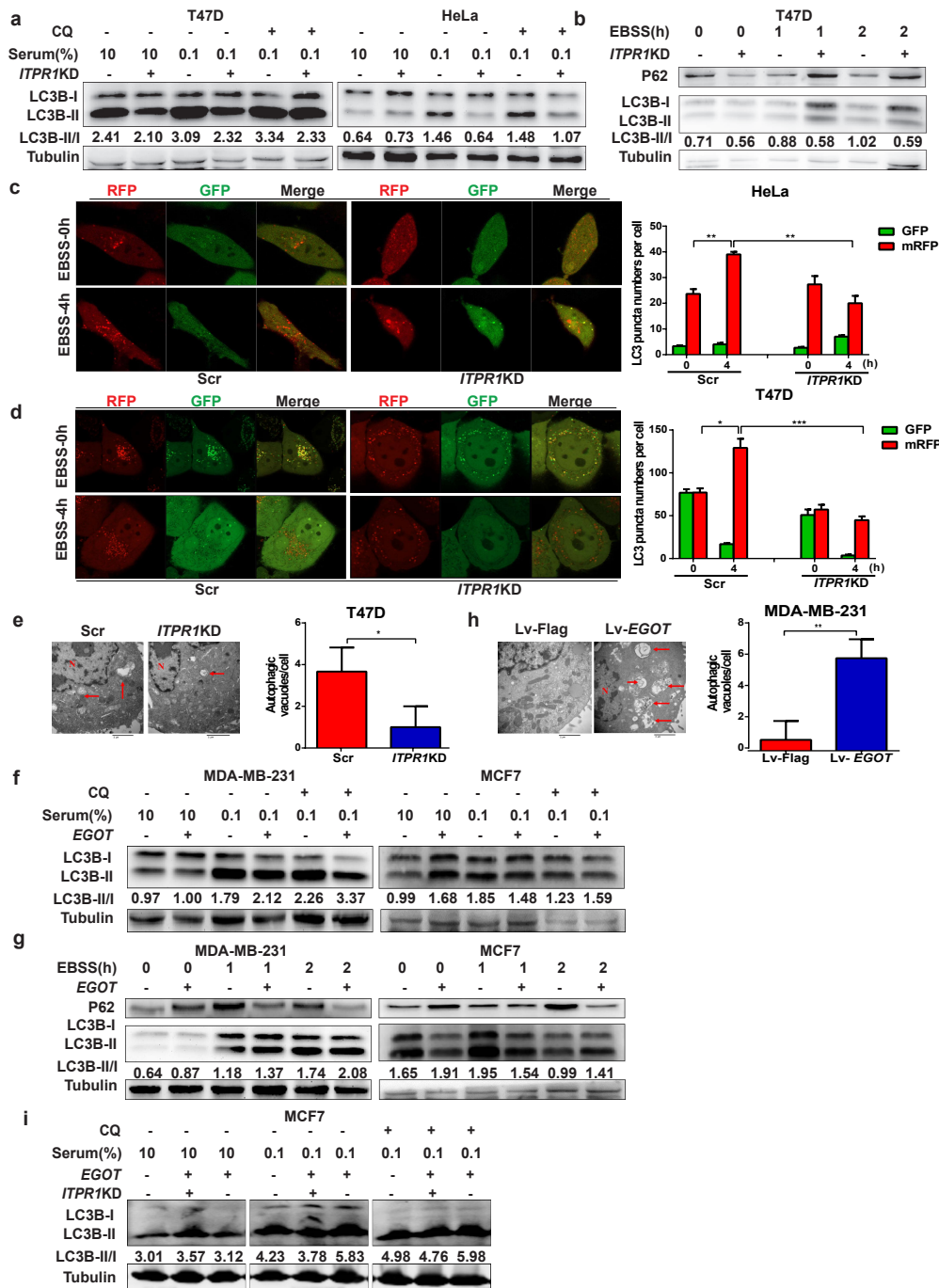

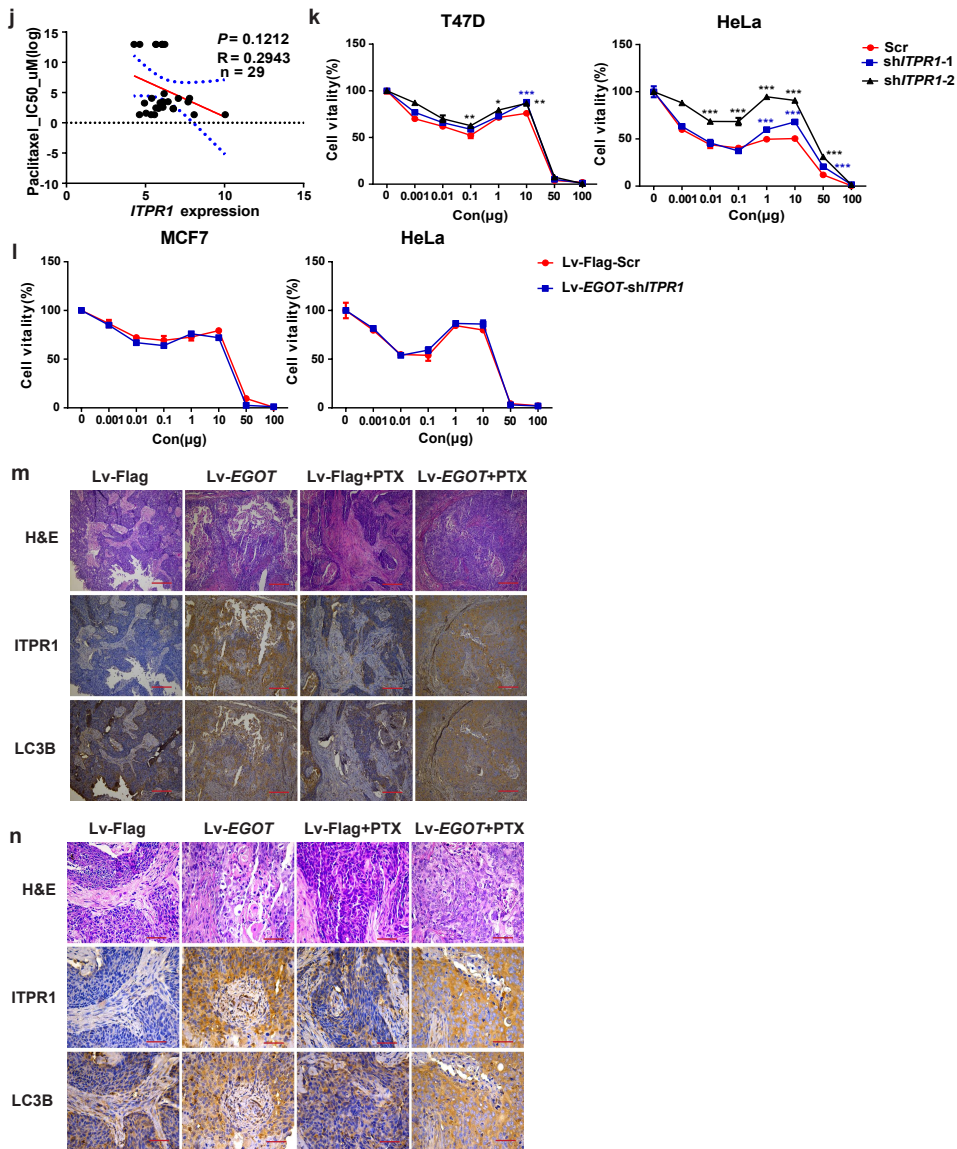

Supplement: Supplementary file 1 — Table S1. Lists of primers. Lists including PCR primers used in this study, primers used for shRNAs and siRNA sequences. Table S2. Details of the gene sequence probe sets. Table S3. Guilt-by-association analysis in breast cancer data from TCGA. Table S4. Pan-cancer data of 33 cancer contexts in TCGA. All cancer IDs and patient numbers are listed. Table S5. Protein mass spectrometry analysis in MDA-MB-231. RNA pull-down assays using the Flag-MS2bp-MS2bs-based system (Additional file 2: Figure S4F), followed by mass spectrometry in MDA-MB-231 cells. Raw data listing all identified proteins and all peptides from each sample. (ZIP 9889 kb) [file 12943_2019_1017_MOESM1_ESM.zip › Figure S3.pdf]

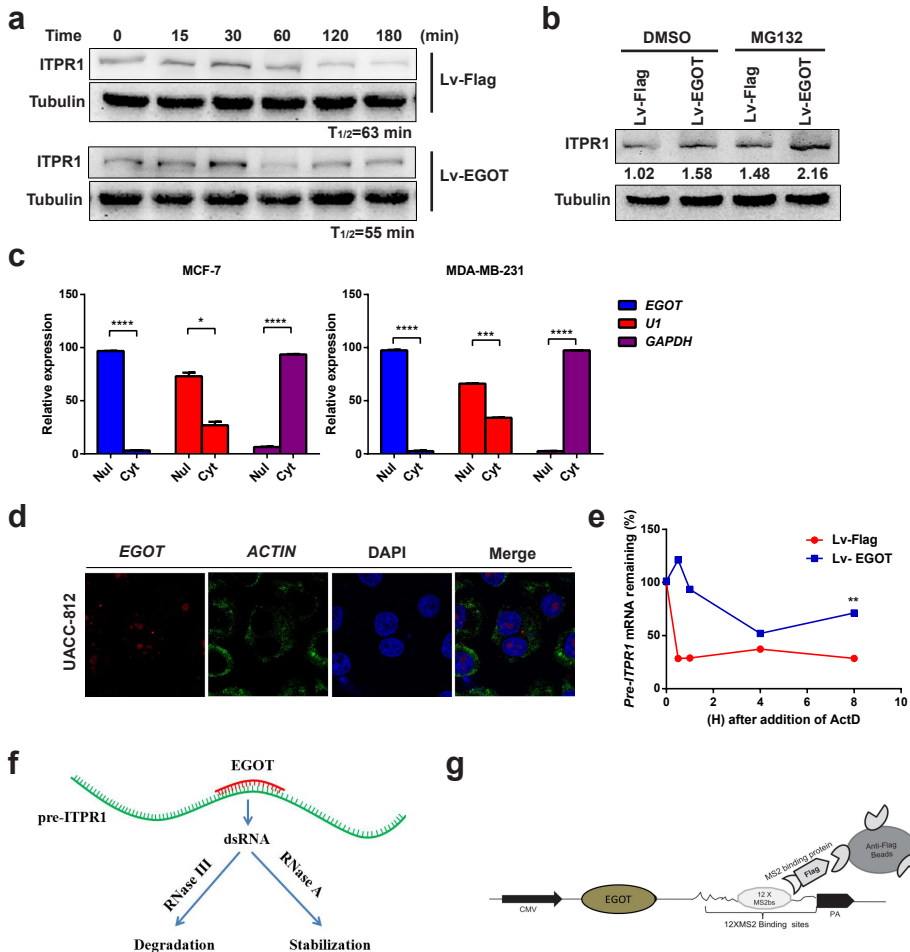

Supplement: Supplementary file 1 — Table S1. Lists of primers. Lists including PCR primers used in this study, primers used for shRNAs and siRNA sequences. Table S2. Details of the gene sequence probe sets. Table S3. Guilt-by-association analysis in breast cancer data from TCGA. Table S4. Pan-cancer data of 33 cancer contexts in TCGA. All cancer IDs and patient numbers are listed. Table S5. Protein mass spectrometry analysis in MDA-MB-231. RNA pull-down assays using the Flag-MS2bp-MS2bs-based system (Additional file 2: Figure S4F), followed by mass spectrometry in MDA-MB-231 cells. Raw data listing all identified proteins and all peptides from each sample. (ZIP 9889 kb) [file 12943_2019_1017_MOESM1_ESM.zip › Figure S4.pdf]

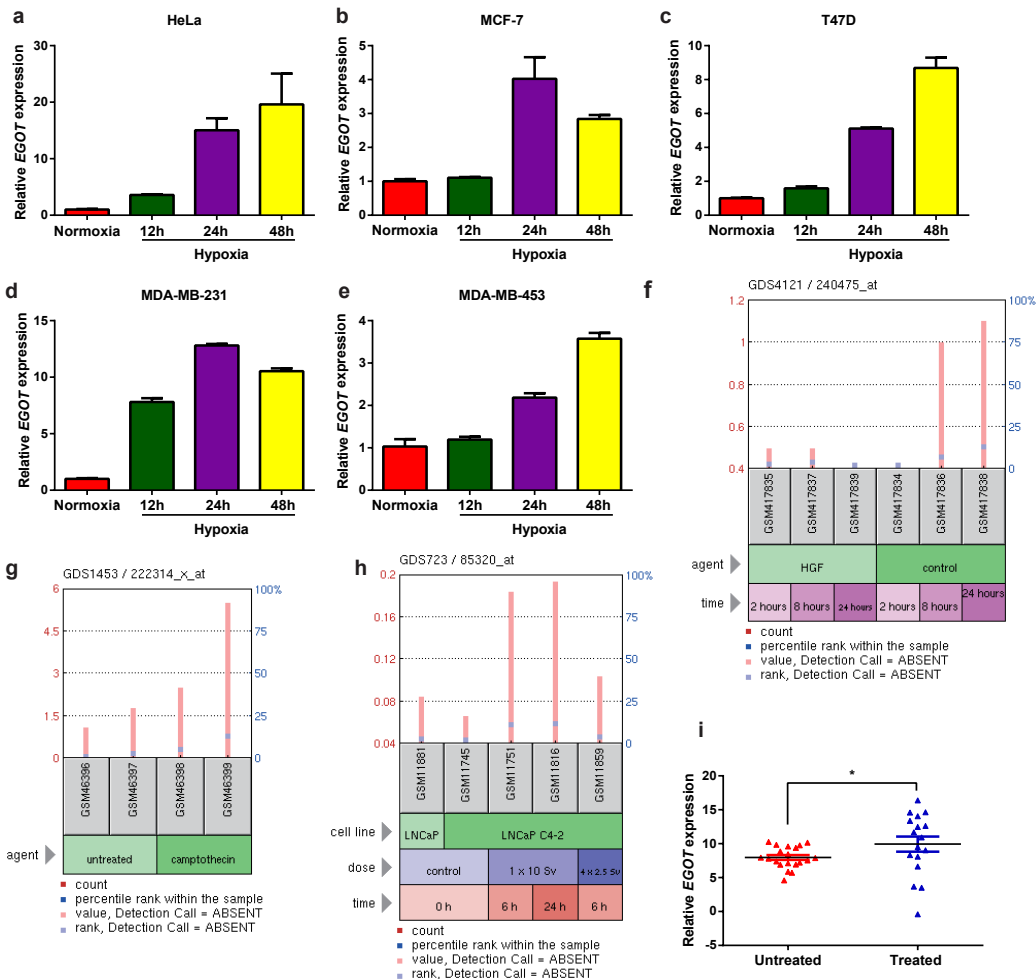

Supplement: Supplementary file 1 — Table S1. Lists of primers. Lists including PCR primers used in this study, primers used for shRNAs and siRNA sequences. Table S2. Details of the gene sequence probe sets. Table S3. Guilt-by-association analysis in breast cancer data from TCGA. Table S4. Pan-cancer data of 33 cancer contexts in TCGA. All cancer IDs and patient numbers are listed. Table S5. Protein mass spectrometry analysis in MDA-MB-231. RNA pull-down assays using the Flag-MS2bp-MS2bs-based system (Additional file 2: Figure S4F), followed by mass spectrometry in MDA-MB-231 cells. Raw data listing all identified proteins and all peptides from each sample. (ZIP 9889 kb) [file 12943_2019_1017_MOESM1_ESM.zip › Figure S5.pdf]

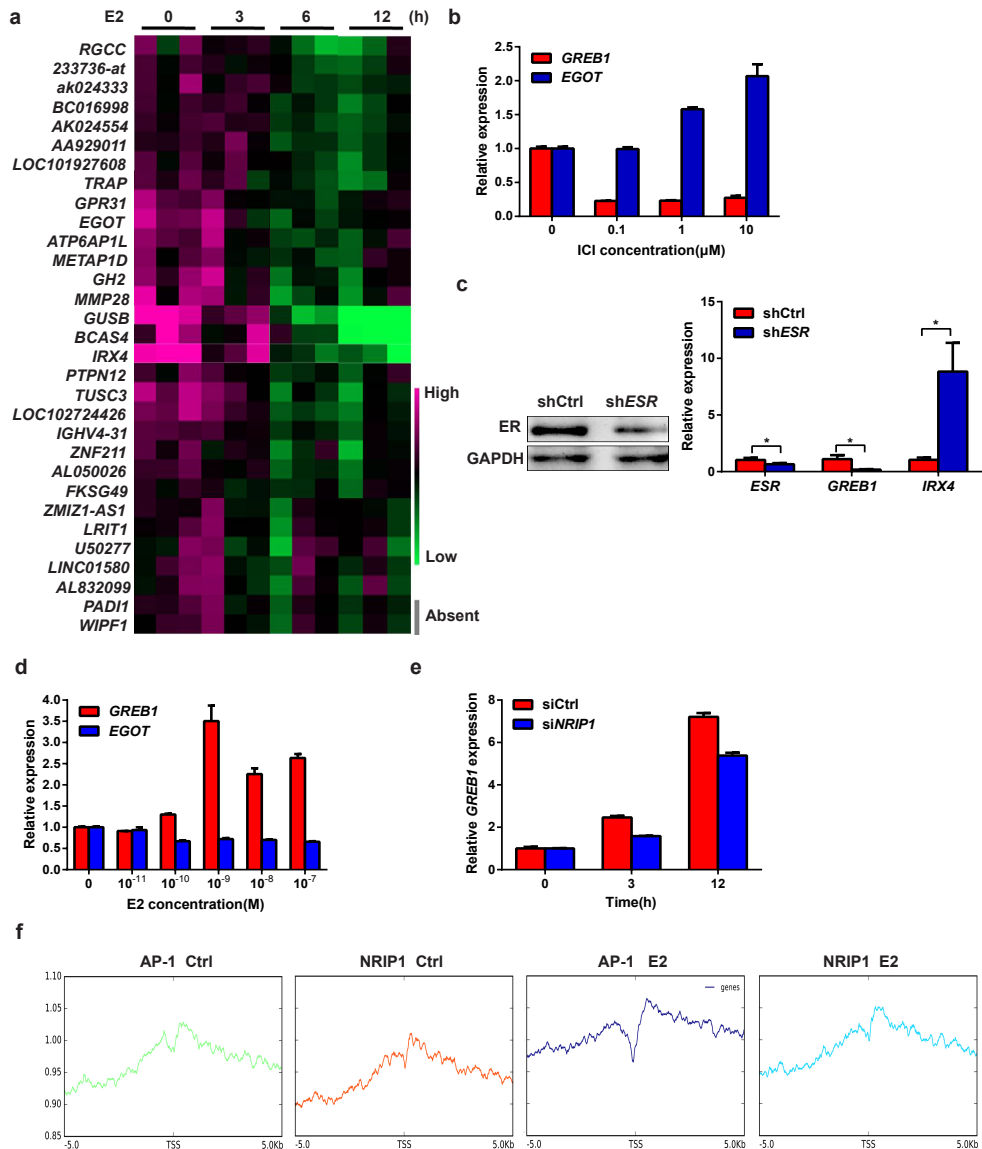

Supplement: Supplementary file 1 — Table S1. Lists of primers. Lists including PCR primers used in this study, primers used for shRNAs and siRNA sequences. Table S2. Details of the gene sequence probe sets. Table S3. Guilt-by-association analysis in breast cancer data from TCGA. Table S4. Pan-cancer data of 33 cancer contexts in TCGA. All cancer IDs and patient numbers are listed. Table S5. Protein mass spectrometry analysis in MDA-MB-231. RNA pull-down assays using the Flag-MS2bp-MS2bs-based system (Additional file 2: Figure S4F), followed by mass spectrometry in MDA-MB-231 cells. Raw data listing all identified proteins and all peptides from each sample. (ZIP 9889 kb) [file 12943_2019_1017_MOESM1_ESM.zip › Figure S6.pdf]

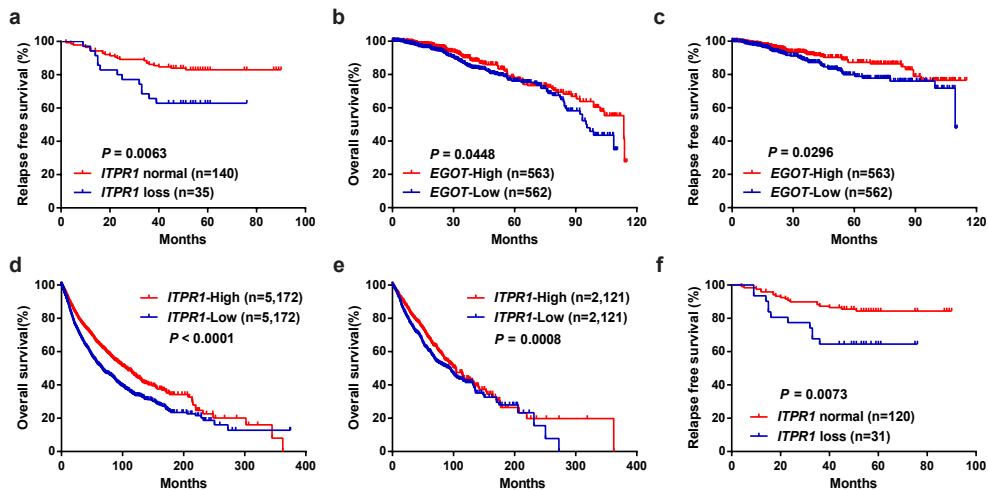

Supplement: Supplementary file 1 — Table S1. Lists of primers. Lists including PCR primers used in this study, primers used for shRNAs and siRNA sequences. Table S2. Details of the gene sequence probe sets. Table S3. Guilt-by-association analysis in breast cancer data from TCGA. Table S4. Pan-cancer data of 33 cancer contexts in TCGA. All cancer IDs and patient numbers are listed. Table S5. Protein mass spectrometry analysis in MDA-MB-231. RNA pull-down assays using the Flag-MS2bp-MS2bs-based system (Additional file 2: Figure S4F), followed by mass spectrometry in MDA-MB-231 cells. Raw data listing all identified proteins and all peptides from each sample. (ZIP 9889 kb) [file 12943_2019_1017_MOESM1_ESM.zip › Figure S7.pdf]

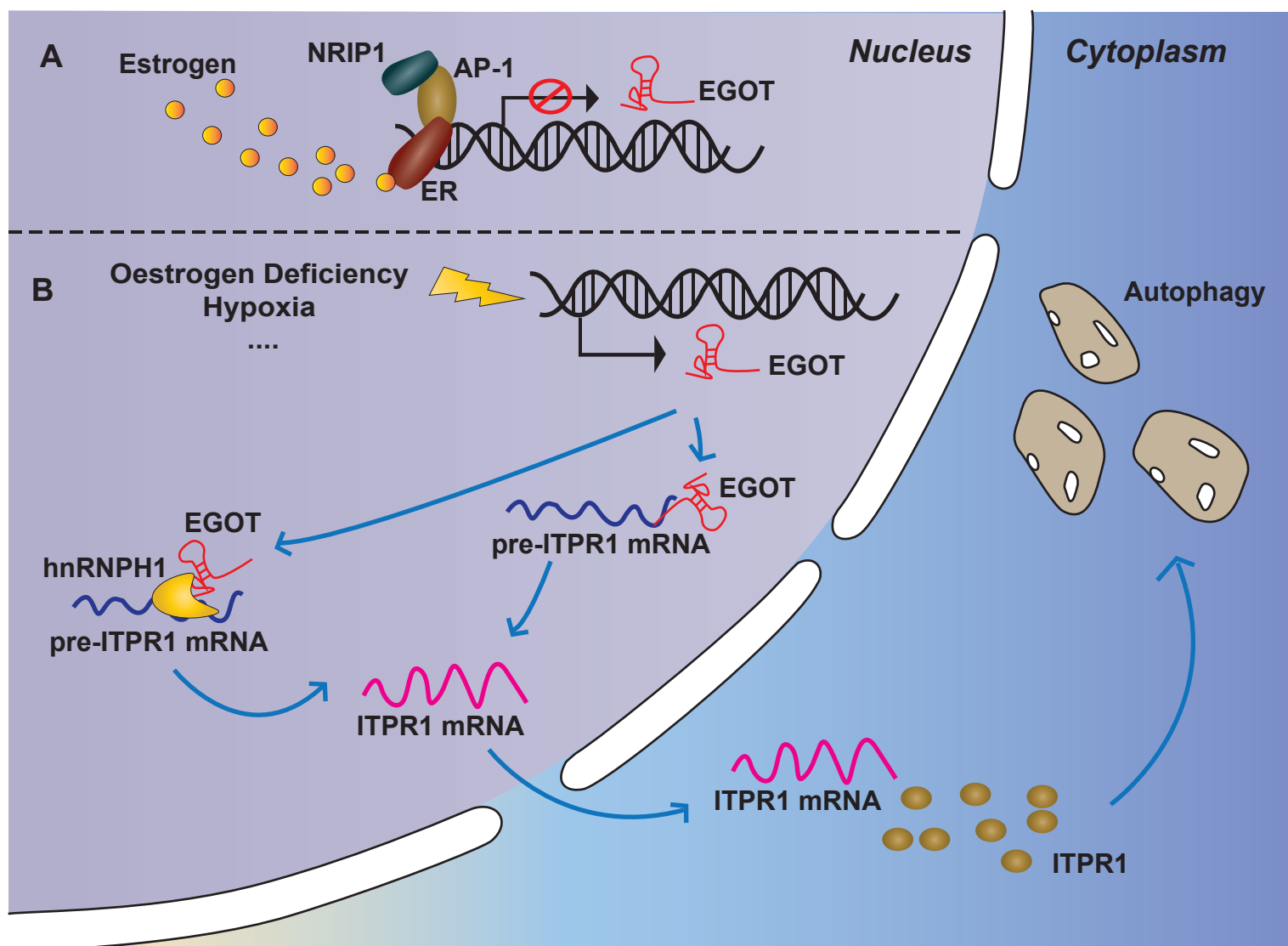

Supplement: Supplementary file 1 — Table S1. Lists of primers. Lists including PCR primers used in this study, primers used for shRNAs and siRNA sequences. Table S2. Details of the gene sequence probe sets. Table S3. Guilt-by-association analysis in breast cancer data from TCGA. Table S4. Pan-cancer data of 33 cancer contexts in TCGA. All cancer IDs and patient numbers are listed. Table S5. Protein mass spectrometry analysis in MDA-MB-231. RNA pull-down assays using the Flag-MS2bp-MS2bs-based system (Additional file 2: Figure S4F), followed by mass spectrometry in MDA-MB-231 cells. Raw data listing all identified proteins and all peptides from each sample. (ZIP 9889 kb) [file 12943_2019_1017_MOESM1_ESM.zip › Figure S8.pdf]
